# Polarisation vision: overcoming challenges of working with a property of light we barely see

**DOI:** 10.1007/s00114-018-1551-3

**Published:** 2018-03-27

**Authors:** James J. Foster, Shelby E. Temple, Martin J. How, Ilse M. Daly, Camilla R. Sharkey, David Wilby, Nicholas W. Roberts

**Affiliations:** 1 Vision Group, Department of Biology, Lund University, Sölvegatan 35, 223 62 Lund, Sweden; 2 Ecology of Vision Laboratory, School of Biological Sciences, Life Sciences Building, University of Bristol, Tyndall Avenue, Bristol, BS8 1TQ UK; 3 Present Address: Azul Optics Ltd., 7 Bishop Manor Road, Westbury-On-Trym, Bristol, BS10 5BD UK; 4 Department of Physiology, Development and Neuroscience, Cambridge University, Cambridge, CB2 3EG UK

**Keywords:** Polarisation, Vision, Methods, Measurement, Imaging, Artefact

## Abstract

In recent years, the study of polarisation vision in animals has seen numerous breakthroughs, not just in terms of what is known about the function of this sensory ability, but also in the experimental methods by which polarisation can be controlled, presented and measured. Once thought to be limited to only a few animal species, polarisation sensitivity is now known to be widespread across many taxonomic groups, and advances in experimental techniques are, in part, responsible for these discoveries. Nevertheless, its study remains challenging, perhaps because of our own poor sensitivity to the polarisation of light, but equally as a result of the slow spread of new practices and methodological innovations within the field. In this review, we introduce the most important steps in designing and calibrating polarised stimuli, within the broader context of areas of current research and the applications of new techniques to key questions. Our aim is to provide a constructive guide to help researchers, particularly those with no background in the physics of polarisation, to design robust experiments that are free from confounding factors.

**Electronic supplementary material:**

The online version of this article (10.1007/s00114-018-1551-3) contains supplementary material, which is available to authorized users.

## Introduction

### The challenge of studying polarisation sensitivity

Polarised light is abundant in nature and, since the discovery of polarisation-sensitive orientation in honeybees (von Frisch 1949), a great number of animal species have been shown to use polarised light to inform their behaviour (Horváth and Varjú 2004). While most research, until very recently, has focused on navigation and orientation behaviours that use wide-field environmental polarisation cues, we are now beginning to uncover the remarkable complexity of the polarisation patterns within visual scenes that many species are able to see and use.

That it has taken us so long to appreciate the value of these polarisation cues and signals, and the sensitivity of animals to them, is no doubt due to the limited polarisation sensitivity of our own visual system. Although, under the correct circumstances, humans can detect and identify polarised light via the Haidinger’s brushes phenomenon (von Haidinger 1844; Shurcliff 1955; Temple et al. 2015), we are ‘polarisation blind’ in our daily lives when compared with the majority of animal species. It has only been with the aid of recently developed polarisation imaging technologies (e.g. Powell and Gruev 2013; Roberts et al. 2014; York et al. 2014; Gagnon and Marshall 2016) that we are beginning to uncover the complexities of the polarisation patterns that exist in nature and, with this, starting to understand how animals use this information.

The aim of this review is to provide a constructive guide for researchers who wish to study polarisation sensitivity, but may be unfamiliar with the terminology, the measurement techniques, the advantages and drawbacks of different means of producing polarised stimuli, and, most importantly, the various pitfalls of intensity and spectral confounds. For researchers who are more familiar with the field, this review provides an up-to-date collection of the various methods developed to address these challenges. More broadly, this review may be used as a guidebook in the early stages of planning a study; to help determine the equipment, measurements, and control experiments necessary. Finally, we hope that the discussions below will inspire new research, lead to further improvements to these methods, and aid in the interpretation of animal polarisation vision experiments.

### Key areas of research

Throughout the animal kingdom polarisation sensitivity is employed for a broad range of functions (reviews: Wehner 2001; Labhart 2016). An understanding of how animals respond to and use the polarisation of light is of importance to researchers interested in their sensory ecology. Key areas of research can be summarised under two broad uses of the polarisation of light: *contrast vision*, where polarisation information is used for object-based visual tasks; and *environmental assessment*, where polarisation cues vary over broad spatiotemporal scales and are used to inform navigation or habitat selection behaviour.

#### Contrast vision

Contrast vision is an important feature of many visual systems and mediates a wide range of perceptual tasks, such as detecting predators, identifying prey, or visual communication. The role of polarisation in these processes is an expanding field of research. One area of focus is the capacity to separate a visual field into objects and background using polarisation, roughly analogous to the use of colour vision for image segmentation (e.g. How et al. 2015). In colour-sensitive animals, polarisation might be used in combination with colour to augment the effectiveness of object detection (e.g. Kelber 1999; Kinoshita et al. 2011), and in colourblind species (such as most cephalopods) polarisation may represent a primary means by which image segmentation is achieved (Cronin et al. 2003). Polarisation sensitivity might also improve object detection efficiency by permitting the removal of object-concealing scattered spacelight from the visual scene (see Sources of Polarised Light—Scattered Light), enhancing visual contrast (Lythgoe and Hemmings 1967; Cartron et al. 2013; Sharkey et al. 2015).

Contrast vision is not limited to object detection. There is also evidence that polarisation-sensitive species of cephalopod mollusc and stomatopod crustacean display polarised body patterns that act as visual signals (Shashar et al. 1996; Cronin et al. 2009; Chiou et al. 2011; How et al. 2014b; Gagnon et al. 2015). In the case of circularly polarised carapace reflections in stomatopods (see Biological Polarizers), these signals would represent a private communication channel that even other polarisation-sensitive animals would be blind to (Chiou et al. 2008; Gagnon et al. 2015). It has also been suggested that flowers may signal their profitability to pollinators via patterns in the reflection of polarised light (Foster et al. 2014) and that plant viruses may manipulate polarisation reflected from leaf surfaces to attract vectors (Maxwell et al. 2016).

A great range of aquatic insect species, and at least one species of aquatic springtail (Egri et al. 2016), are thought to identify bodies of water from the horizontally polarised light reflected at the water’s surface (Schwind 1983, 1989, 1995; Horváth and Kriska 2008). Horizontally polarised light also triggers oviposition behaviour in swallowtail butterfly *Papilio xuthus*, which uses the angle of polarisation of reflected light in combination with colour cues to detect appropriate leaves for oviposition (Kelber 1999; Kinoshita et al. 2011). This is a good example of how polarised light may provide additional information about a visual object once it has been detected.

#### Environmental assessment

Polarised light may also provide information about more broad-field environmental cues than those mentioned above. The best studied example is the incorporation of information from polarised skylight into the celestial compass of many insect species (reviews: Wehner 2001; Horváth and Varjú 2004; Horváth et al. 2014), a capacity that has also been suggested in some crustacean (Bainbridge and Waterman 1957) and mollusc species (Jander et al. 1963), and even some vertebrates (Taylor and Adler 1973; Able and Able 1993; Parkyn et al. 2003). Since the pattern of polarised skylight indicates the sun’s compass bearing (the solar azimuth), it may be used as a reference frame for geographic body-axis orientation when the sun is not visible. Many insect species possess a specialised polarisation-sensitive region in the dorsal eye, the dorsal rim area (DRA), which is used to detect this skylight pattern (Wehner and Strasser 1985; Labhart and Meyer 1999). At night, when scattered sunlight is no longer present, moonlight scattered via the same process provides an equivalent polarisation pattern (Gál et al. 2001) that can be used by crepuscular and nocturnal dung beetles, and perhaps other night active insects, for orientation (Dacke et al. 2004; el Jundi et al. 2015).

In aquatic environments, polarisation may also provide information about water depth. A study involving water flea *Daphnia pulex* found that, when presented with a choice of two polarised light fields, this species is attracted towards the more strongly polarised stimulus (Schwind 1999). This behaviour is suggested to achieve ‘shore flight’ towards deeper water (which often produces more strongly polarised spacelight) and away from shore-dwelling predators. Since polarised spacelight is a feature of many aquatic habitats (see Scattered Light), other aquatic species may also use polarised light to guide their shore-flight responses.

While hypotheses on the function of both polarisation contrast vision and the use of polarised environmental cues have been under study for some time, at the time of writing both are reaching a new period of improved characterisation and understanding. Recent innovations in the use of Liquid Crystal Displays (see Twisted Nematic Liquid Crystal Displays and Patterned Vertical Alignment Liquid Crystal Displays) have led to clear demonstrations of the extraordinary polarisation vision of cephalopods and crustaceans (Temple et al. 2012; How et al. 2014a; Daly et al. 2016). Careful study of the neuronal processing of skylight polarisation cues in insects has led to an improved model for how skylight polarisation is interpreted (Pfeiffer and Homberg 2007; Bech et al. 2014) and combined with other orientation cues (el Jundi et al. 2015). As we come closer to understanding the details of this aspect of the animal visual world that remains alien to our intuition, it continues to be important to focus on the methods we use to produce, control and measure polarised stimuli.

## Polarised light

### What is polarised light?

#### Light—an electromagnetic wave

Light is a form of electromagnetic radiation, part of a spectrum that includes X-rays, microwaves and radio waves. In general, it passes through space as a *transverse* wave (i.e. oscillating at right angles to the direction of travel) that consists of an oscillating electric and magnetic field. For biological systems, we usually only consider the orientation of the electric field, since it is this that directly affects light absorption, and hence vision. Any electromagnetic wave also consists of discrete quantized packets of energy, called photons. Bright light contains more photons than dim light, and the energy of the wave is proportional to the frequency of the light.

**Fig. 1:**
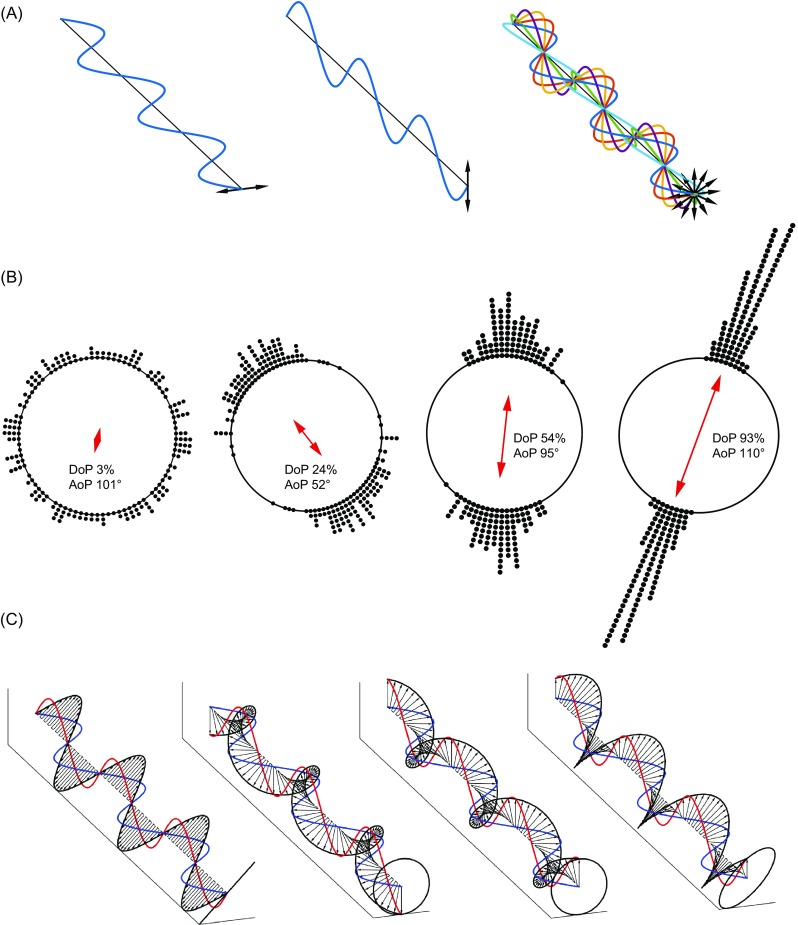
Visualising polarisation states. **a** Three beams of light propagating towards us along the same axis. Each has a different polarisation state. The left and centre panels show 100% horizontally and vertically polarised light. To the right is 0% polarised (unpolarised) light comprising waves that oscillate with a uniform distribution of angles. Shown as a coherent, monochromatic beam to aid visualisation (most light is incoherent: the waves do not have a defined relationship with one another). In (**b**) points on the outside of the circle represent randomly sampled angle distributions of a series of waves comprising a beam, and the arrow within the circle gives the resultant angle of polarisation. If the constituent waves oscillate in all directions, the beam is unpolarised, degree of polarisation ≈ 0 (left). The beam is partially polarised if the distribution of oscillation planes has an overall direction—its angle of polarisation (centre). The degree of linear polarisation describes the spread in values (precision of their centre). If all waves oscillate in the same plane, the light is completely linearly polarised: degree of polarisation ≈ 1 (right). (**c**) Circular polarisation and ellipticity, in waves shown as being made up of a vertical (red) and horizontal (blue) component. Ellipticity is governed by the relative phase (distance between peaks) between these two components. A phase difference of zero (or integer multiple of a half wavelength) results in (diagonally) linearly polarised light (left). Phase differences of a quarter of a wavelength give left-handed (left-centre) or right-handed (right-centre) circularly polarised light. Phase differences between these two limits give elliptically polarised light (right). In these example cases, the components’ amplitudes are identical

Polarisation is, however, a distinct property of light that defines both the relative orientations of the waves as they propagate and how light is reflected, refracted, scattered and transmitted by different materials. A beam of light usually consists of a large number of waves, and the polarisation of a light source concerns the distribution of the orientation of the electric fields of the waves. A beam in which all of the waves oscillate horizontally is called fully horizontally polarised light (Fig. 1a, left). If all of the waves in the beam oscillate vertically then it is fully vertically polarised (Fig. 1a, middle). The predominant axis of the distribution of the waves from a light source is the *angle of polarisation* (AoP). The AoP is an angular measure that can vary between 0° and 180°. A second property is the *degree of polarisation* (DoP; or percent polarisation) and this is the ratio of the (average) intensity of the polarised portion of the beam to its total (average) intensity. For example, unpolarised light, which is composed of multiple waves with a uniform distribution (Fig. 1a right) has a DoP of 0, while plane (or linearly) polarised light, in which all waves are oscillating in a single plane, has a DoP of 1. Natural scenes tend to contain light with DoP values ranging between 0 and around 0.5.

The property of *ellipticity* is less straightforward to understand. By describing a wave of light as being composed of two components with perpendicular electric fields, the ellipticity is a measure of the phase relationship and the relative amplitudes of these two components. At any time point, the distribution of the resultant electric field of a beam of light maps out as an ellipse, or in one limiting case, a circle for circularly polarised light (Fig. 1c). Most light in nature is not elliptically polarised and the vast majority of animal eyes do not analyse the ellipticity of polarised light (although mantis shrimps provide an exception in both of these cases; see Biological polarisers). Because there is no difference between the probability of absorbance of either handedness (rotation direction) of circularly polarised light by visual pigment chromophores, the ellipticity has no effect on the detection of polarised light at the photoreceptor level. This is encapsulated by the measurement of the *degree of linear polarisation* (DoLP; see Table 1), which disregards any elliptical component.

**Table 1:** Describing polarisation

Angle of polarisation (AoP; also commonly (but incorrectly) referred to as the e-vector angle or χ)	Describes the predominant angle (relative to some external reference e.g. horizontal or vertical) along which the electric fields in a light beam oscillate. While this is often referred to as the e-vector angle, the term is not entirely appropriate, since the angle of the electric field vector is a property of single wave and not of a time-averaged beam of light. AoP should not be confused with the polarising angle, which describes the angle of incidence at which an unpolarised beam of light becomes maximally polarised after reflection from a surface (see Surface Reflections).
Circular polarisation and elliptical polarisation	Best described by a wave made up of two electric field components (Fig. 1c) directed vertically (red) and horizontally (blue). If their points of maximum and minimum amplitude (peaks and troughs) are aligned (in phase; Fig. 1c, left) the resultant electric field oscillation is linear. When these points are out of phase, the resultant electric field traces an ellipse. If this phase difference is a quarter of a wavelength (π/2) then the electric field vector traces a corkscrew of circular cross-section: circularly polarised light. Any phase difference not an integer multiple of π/2 (or 0) results in elliptically polarised light. Note however this is only a model to aid our interpretation of observed phenomena.
Degree of polarisation (DoP; also commonly referred to as percent polarisation or ẟ)	Refers to the proportion of waves in a source of light that have a particular polarisation state. DoP varies between 0 (for unpolarized light) and 1 (for completely polarised light). DoP accounts for linear and elliptically polarised light and is calculated from the Stokes parameters (see below). If light has an elliptical component, degree of linear polarisation (DoLP) may describe its discriminability for a polarisation-sensitive animal better than DoP, since light that is highly linearly polarised or highly elliptically polarised both have high DoP, but the elliptical component must be converted to linear before it can be discriminated.
Degree of linear polarisation (DoLP)	Describes the proportion of waves in a light source that are oriented in a particular plane. To determine how a light source appears to an animal with linear polarisation vision, the DoLP is a practical, relevant measure.
Optic axis, fast and slow axis	For a material with more than one refractive index, the optic axis is the axis along which light can propagate, and, irrespective of the angle of polarisation, only see (be affected by) one of those refractive indices. The slow axis (Fig. 1c, red: left centre, blue: right centre) is the orientation for which the polarisation is slowed the most by seeing the highest refractive index. Conversely, the fast axis is the orientation for which light sees the lower refractive index. Retardation, and the conversion of one form of polarisation to another (e.g. linear polarised light becoming circular) occurs when the angle of polarisation is not coincident with either the fast or slow axis of the material. The resolved components therefore travel at different speeds and a phase difference between the components is introduced.
Refractive index	The ratio of the speed of light in a vacuum to its speed in a material. The speed of light in many materials depends on the substance’s orientation, and many crystals have two or three different refractive indices depending on a wave’s angle of polarisation relative to the crystal axes.
Transmission axis (TA)	The axis parallel to the angle of polarisation transmitted by a linear polarizer. The ratio of transmission through two polarizers with perpendicular or parallel transmission axes gives their extinction ratio.

#### Stokes parameters

Stokes parameters are a mathematical representation of the polarisation of light and are often used to calculate the AoP, the DoP and ellipticity. By setting *I* as the value of the total light intensity and letting *I*_α_ represent the intensity of light that is transmitted through a polarizer with a transmission axis orientated at α, the Stokes parameters (S_0_, S_1_, S_2_, S_3_), can be defined as

1 $$ {\mathrm{S}}_0=I $$

2 $$ {\mathrm{S}}_1={I}_0-{I}_{90} $$

3 $$ {\mathrm{S}}_2={I}_{45}-{I}_{135} $$

4 $$ {\mathrm{S}}_3={I}_{\mathrm{Left}}-{I}_{\mathrm{Right}}. $$

While S_1_ and S_2_ provide information on linear polarisation, S_3_ provides a measure of the ellipticity, calculating the difference between the left-handed, *I*_Left_, and right-handed, *I*_Right_, components. Note that S_0_ is the total light intensity.

The angle of polarisation, AoP, is given by

5 $$ \mathrm{AoP}=\frac{1}{2}\arctan \left(\frac{{\mathrm{S}}_1}{{\mathrm{S}}_2}\right) $$

and the degree of polarisation, DoP, is given by

6 $$ \mathrm{DoP}=\frac{\sqrt{{{\mathrm{S}}_1}^2+{{\mathrm{S}}_2}^2+{{\mathrm{S}}_3}^2}}{{\mathrm{S}}_0}. $$

The degree of linear polarisation, DoLP, is given by

7 $$ \mathrm{DoLP}=\frac{\sqrt{{{\mathrm{S}}_1}^2+{{\mathrm{S}}_2}^2}}{{\mathrm{S}}_0} $$

and the ellipticity is given by

8 $$ \epsilon =\frac{{\mathrm{S}}_3}{{\mathrm{S}}_0}. $$

For details of the measurement systems that can be used to calculate the Stokes parameters of stimulus light, see How Polarised Light is Measured.

### Sources of polarised light in nature

Many habitats are rich in polarisation information. It is therefore important to consider sources of polarised light in an animal’s natural environment when designing or interpreting a study of polarisation sensitivity.

#### Scattered light

The most broad-field sources of polarised light in nature are scattered skylight and aquatic spacelight. Through the process of Rayleigh scattering (after: Strutt 1871) sunlight entering the upper atmosphere is scattered towards a terrestrial observer by scattering centres (atoms, molecules, aerosols, air density fluctuations) smaller than the wavelength of light. The DoP of this scattered skylight increases as a function of the angular deviation from its original path, reaching a maximum at 90°. Additionally, the AoP of this light is at right angles to the initial path, a consequence of the transverse nature of the electric field. Since the angular deviation required for light from the sun to be scattered towards an observer is different in different parts of the sky, the polarisation state also differs. The result is a pattern in both DoP and AoP across the sky that can be used, in combination with information about time of day, to determine the sun’s position; a reliable orientation reference when the sun itself is not directly visible (Horváth et al. 2014; Wang et al. 2016). When the moon is the primary source of light within the celestial hemisphere, it too creates a pattern of polarised skylight that can be used as an orientation cue (Gál et al. 2001; Dacke et al. 2004).

In aquatic environments, sub-wavelength diameter particles suspended in the water column scatter polarised spacelight towards an observer from object-free space within the environment, termed veiling light when it comes between the viewer and a target. Polarised spacelight creates a pattern of polarisation, surrounding an underwater viewer, with a high DoP band perpendicular to the predominant direction of downwelling light that tilts with the sun’s elevation (Cronin and Shashar 2001). It has been proposed (Lythgoe and Hemmings 1967) that polarisation-sensitive aquatic species might filter out background spacelight (Johnsen et al. 2011) or veiling light (Cartron et al. 2013; Sharkey et al. 2015) using its polarisation, thereby increasing object-background contrast.

Scattered skylight and spacelight represent the most common sources of polarised light in nature; skylight is available to animals in most environments and a variety of aquatic environments permit access to both. The broad-field nature of these cues, and their patterns in angle and degree of polarisation, mean that they can be challenging to replicate under laboratory conditions. Often a large sheet of polarizer can suffice in eliciting orientation behaviour similar to that under natural skylight, or simulate a background of polarised spacelight in an aquarium setting, but see the sections regarding liquid crystal displays, scattering media and projected polarisation for methods that may be sufficiently versatile to replicate broad-field polarisation patterns more precisely, and the description of viewing-angle effects in polarizers for potential drawbacks of polarising filters as broad-field stimuli.

#### Surface reflections

Specular reflections from most flat surfaces found in the natural world are partially polarised as a function of angle. Particularly prominent are reflections from the surfaces of bodies of water, and as a result many aquatic insects (Horváth and Kriska 2008) and perhaps some other arthropods (Egri et al. 2016), use polarised reflections to identify their habitats. The leaves and petals of plants (Kelber 1999; Kinoshita et al. 2011) and the exoskeletons of arthropods are other smooth surfaces that produce polarised reflections.

Although most materials found in a laboratory can be used as surfaces that produce polarised reflections, they are not necessarily straightforward to manipulate in terms of their polarising properties (see Manipulating Angle of Polarisation—Specular Reflections). Somewhat counterintuitively, a polarising filter may function equally well in eliciting polarotactic behaviour in animals searching for bodies of water or smooth leaves, and allow the experimenter to manipulate the angle (e.g. Kelber 1999) or degree of polarisation (Egri et al. 2016) without affecting the spectrum or intensity of stimulus light.

#### Biological polarisers

**Fig. 2:**
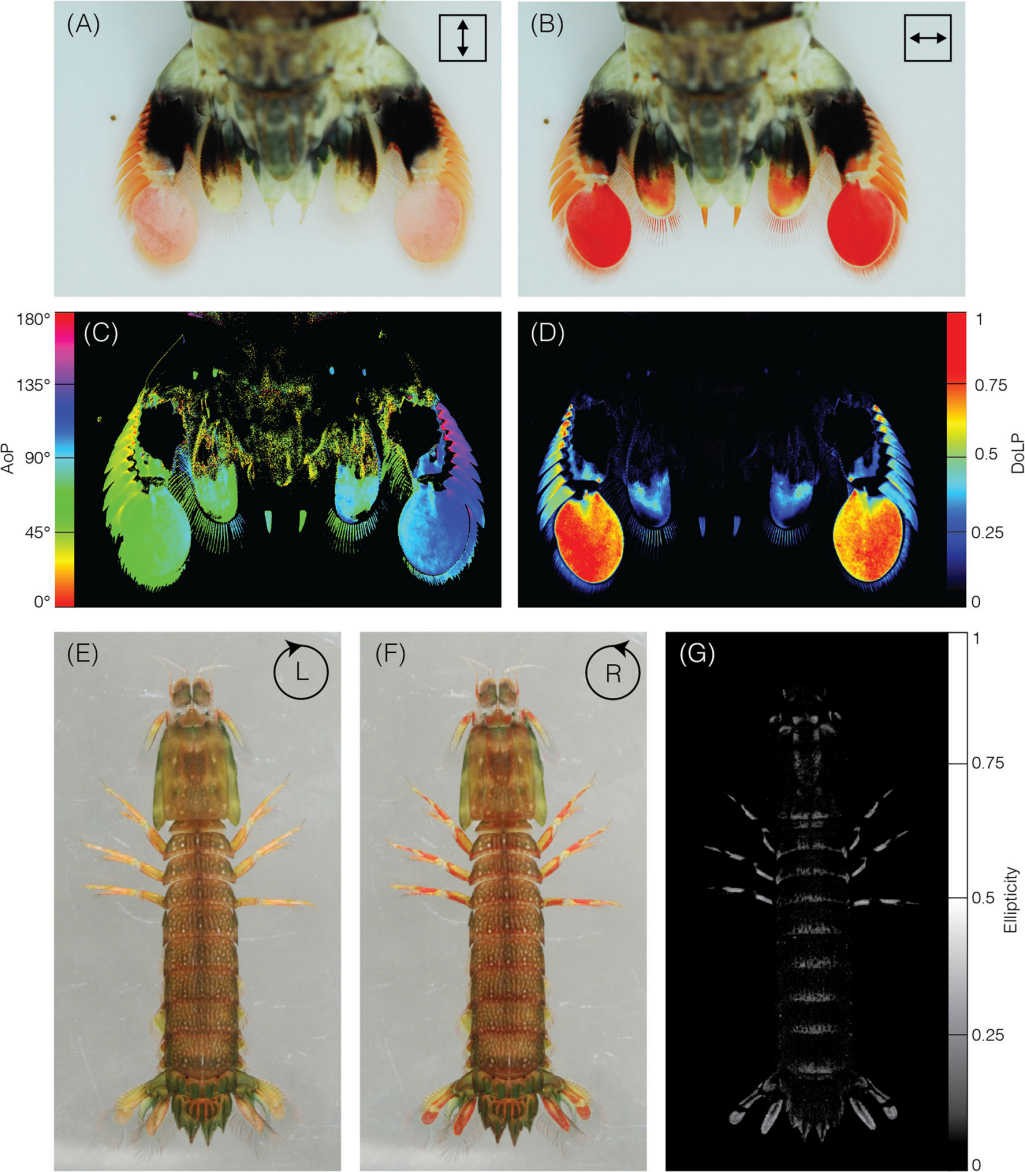
Polarising structures in stomatopods. **a**, **b** Structures on the telson of *Odontodactylus latirostris* linearly polarise reflected and transmitted light. Full colour images recorded through a vertically (**a**) and a horizontally oriented (**b**) linear polarizer, showing the long-pass filtering of horizontally, but not vertically polarised light (red pigment). **c** Angle of polarisation as calculated from a digital camera’s green channel (see Photographic Polarimetry), indicating that, at these wavelengths, light reflected from and transmitted through the telson is horizontally polarised. **d** Degree of linear polarisation across the telson, colour codes shown in adjacent colour bars. **e**, **f** Colour images of *Gonodactylaceus falcatus* recorded through a left-handed and right-handed circular polarizer, highlighting structures on the legs and telson that polarise light with a high degree of ellipticity (**g**)

In addition to environmentally available polarised light, some species are endowed with structures that control the polarisation of light reflected from them (review: Marshall et al. 2014). For instance, many stomatopod crustaceans possess specialised regions in their carapaces that polarise light (Fig. 2). Appendages such as the antennal scales and the first pair of maxillipeds make use of the optical properties of highly ordered molecules of astaxanthin and other photonic structures, respectively, to manipulate the degree and angle of polarisation of the light that is reflected (Cronin et al. 2009; Chiou et al. 2011, 2012; Jordan et al. 2016). Additionally, structures that preferentially reflect circularly or elliptically polarised light (see Table 1) have been found on the carapaces of several species of stomatopod, presumably acting as signals for stomatopod species sensitive to circularly polarised light (Chiou et al. 2008; Gagnon et al. 2015). Polarised reflections may prove to be a widespread feature of stomatopod communication. Many insects also reflect circularly polarised light (e.g. Arwin et al. 2012), however since the discovery by Michelson (1911) over 100 years ago, there still remains very little evidence to suggest any ecological relevance.

The iridophores of cephalopod molluscs, such as the cuttlefish *Sepia officinalis* and the squids *Loligo pealei* and *Euprymna scolopes* also reflect light with a higher DoLP than reflections from other parts of the body, forming patterns that can be concealed or revealed via nervous control of the overlying chromatophore layer (Shashar et al. 1996; Shashar and Hanlon 1997; Chiou et al. 2007; Wardill et al. 2012). Since polarisation sensitivity is widespread in cephalopods, it is likely that these too act as polarised signals. Conversely, silvery fishes such as Atlantic herring, *Clupea harengus,* and European pilchard, *Sardina pilchardus,* incorporate a polarisation preserving optical mechanism in their skin that, unlike most shiny surfaces in nature (see above), does not polarise light via specular reflection at oblique angles. This structure effectively conserves the polarisation of an incident beam as it is reflected from the fish’s skin (Jordan et al. 2012; Jordan et al. 2014), helping to better match the intensity of the background illumination.

While visual signals are often highly context dependent, attempts to artificially manipulate (Boal et al. 2004; Gagnon et al. 2015) polarised signals in the laboratory have met with some success, eliciting responses in both cephalopods and stomatopods. Tests of “polarisation crypsis”, or lack thereof, in silvery fishes may be carried out via photographic polarimetry (see How Polarised Light is Measured) or by observing the behaviour of polarisation-sensitive predators under controlled conditions (e.g. Shashar et al. 2000), although neither approach is straightforward and the application of both methods has met with some criticism (Johnsen et al. 2016).

In general, potential sources of polarisation in an animal’s natural environment should be considered when designing experiments in which animals are exposed to polarised light. For a stomatopod that displays green polarised light while defending its burrow, a blue polarised stimulus may be uninteresting. For an insect that orients using polarised skylight, a polarised stimulus that fills only a small portion of its visual field may not elicit orientation behaviour (Henze and Labhart 2007). To match laboratory stimuli to their presumed natural counterparts, they need to be designed and measured carefully. A comprehensive summary of the best available measurement techniques can be found in the next section.

## How polarised light is measured

As any vision scientist will attest, our own eyes can be unreliable for understanding the properties of light. There are several techniques for measuring the polarisation of light, some of which are robust enough to be performed in any lab or field setting. Here we describe the basic principles behind these techniques and explain some of the advantages and disadvantages of each approach.

**Fig. 3:**
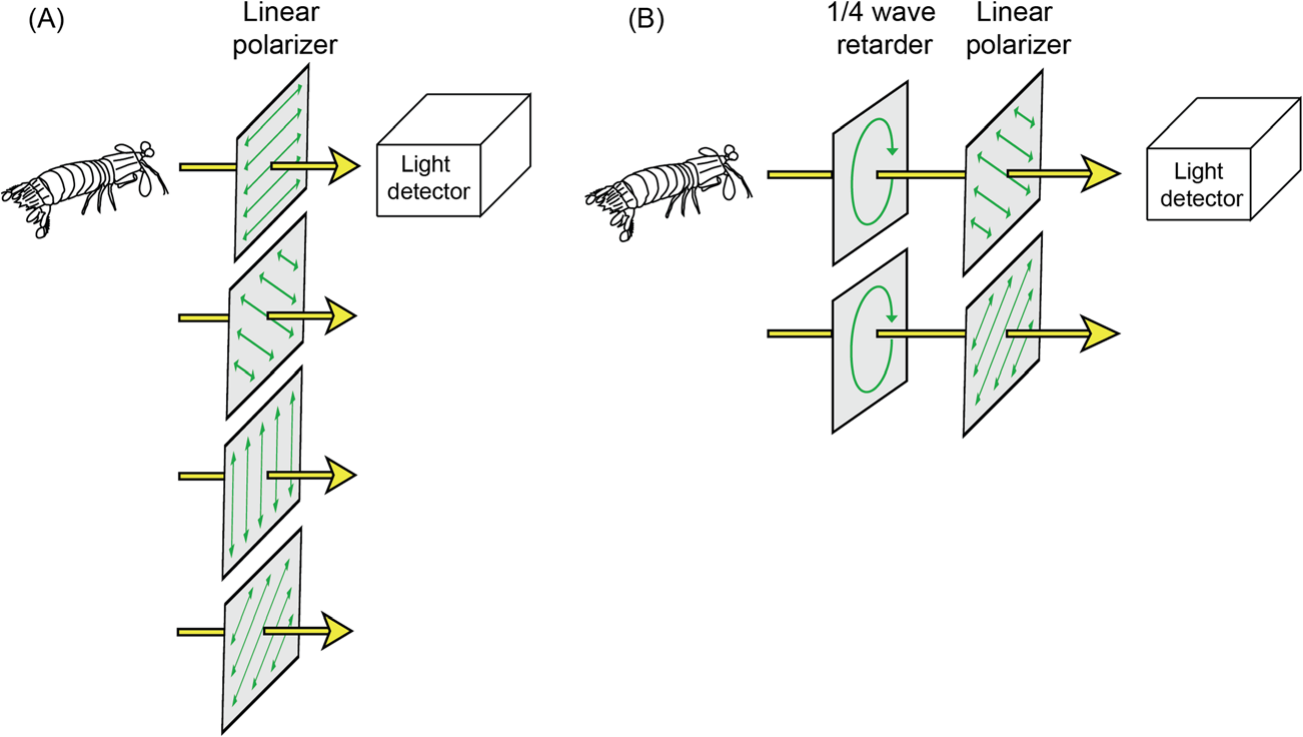
A basic system for measuring the polarisation of light. **a** For measuring Stokes parameters S_0_–S_2_ the measurements are made with the polarizers oriented at 0°, 45°, 90° and 135°. **b** For measuring Stokes parameter S_3_ a combination of a quarter wave retarder and a polarizer at 45° and 135° is used

The most common method for measuring polarisation requires a light detector, light-guiding optics, and a set of polarisation filters (Fig. 3). The polarisation filters convey polarisation sensitivity to the light detector, which is otherwise insensitive to the angle or degree of polarisation (but see supplement S2). A series of spatially or temporally segregated measurements are then taken through filters of different orientations, and these are fed into simple mathematical formulae to extract meaningful polarisation information (see Stokes Parameters).

### Components of a measurement system

#### Light detectors

There are a number of different light detector systems suitable for different kinds of polarisation analysis, including photodiodes and light-sensitive chips (e.g. CCD or CMOS), and the choice of approach is generally determined by what information is needed. A detector may be chosen to provide accurate information across the whole spectrum (Spectral Characterisation), or to approximate the visual sensitivity of the animal in question (Simulated Photoreceptors). In most biological situations, the former is the most desirable method, as few visual systems are well characterised. Furthermore, full spectral measurements of polarisation can always be subsequently transformed to model an animal’s spectral sensitivity or used to check for possible artefacts in other parts of the spectral range. However, some situations, such as sky polarisation sensors, may benefit from a monochromatic measurement system similar to that employed by animals for the same task, through a reduction in the complexity and variability of the information recorded.

In both cases, there are a number of principles that are important to bear in mind. Firstly, polarisation can vary as a function of wavelength, so the spectral sensitivity of the detector is a critical factor. Secondly, most detectors are not linearly sensitive across their stated light intensity range, particularly at the lower and upper limits of this range. This is important given that polarimetry ultimately relies on measuring differences in light intensity (see Stokes Parameters), but can be resolved through calibration. Thirdly, any automatic light adaptation or post-processing mechanisms in the detector must be deactivated to ensure that measurements accurately reflect changes in light levels. Fourthly, it is important to check that none of the optical elements involved in guiding light onto the sensor affect polarisation. For example, since diffraction gratings inside spectrometers diffract light differently depending on its polarisation state (see supplement S2), light needs to be depolarised before entering the spectrometer, and the same is true of some other optical elements. Lastly, when multiple measurements are taken in series, the device and the light environment to be sampled need to remain as static as possible. Any motion or changing light conditions induce intensity changes unrelated to polarisation, resulting in erroneous measurements (so-called motion or temporal artefacts).

#### Polarisation filters

Because very few man-made light detectors are polarisation sensitive, polarimetry methods rely on a series of polarisation filters to differentially absorb specific polarisation components. The minimum requirement for measuring the linear component of polarisation is a single linear polarisation filter, which screens out any light polarised perpendicularly to the transmission axis of the filter, and can range from flexible plastic laminate sheets (see Sheet Polarizers), through to high extinction-ratio calcite polarizers, such as Glan-Thompson polarizers.

#### Retardation devices

To measure the ellipticity of a polarised stimulus, we must add a retardation device. These come in the form of quarter-wave retarders, which are effective at a single wavelength, and Fresnel rhomb retarders, which are effective across a broad spectrum. While quarter-wave retarders convert circular polarisation to linear polarisation by slowing one component by one quarter of a wavelength relative to the other component (see Table 1), Fresnel rhombs exploit a different optical mechanism and use the phase change in the light that takes place upon reflection.

There are two important factors to bear in mind when choosing the appropriate polarisation filters for polarimetry. Firstly, polarisation filters and retardation devices are only effective over specific spectral ranges. In particular, many plastic polarizers do not transmit or polarise well in the UV. A different type of polarizer, known as a wire-grid polarizer, is designed for use in the UV, and the calcite Glan-Thompson type polarizers also have better transmission characteristics. Secondly, most of these filters and retarders only perform optimally when the incident light is normal to the surface of the device; off-axis light will usually induce some level of intensity artefact (see Viewing-Angle Effects in Polarizers); Glan-Thompson polarizers also have a limited acceptance angle. These two factors are relatively straightforward to measure and many manufacturers provide this information with their products.

Here we describe three basic systems that biologists may find useful for measuring polarisation. Each has its own advantages and limitations, and each can be modified or enhanced in different ways to improve its effectiveness.

### Example systems

#### Photographic polarimetry

**Fig. 4:**
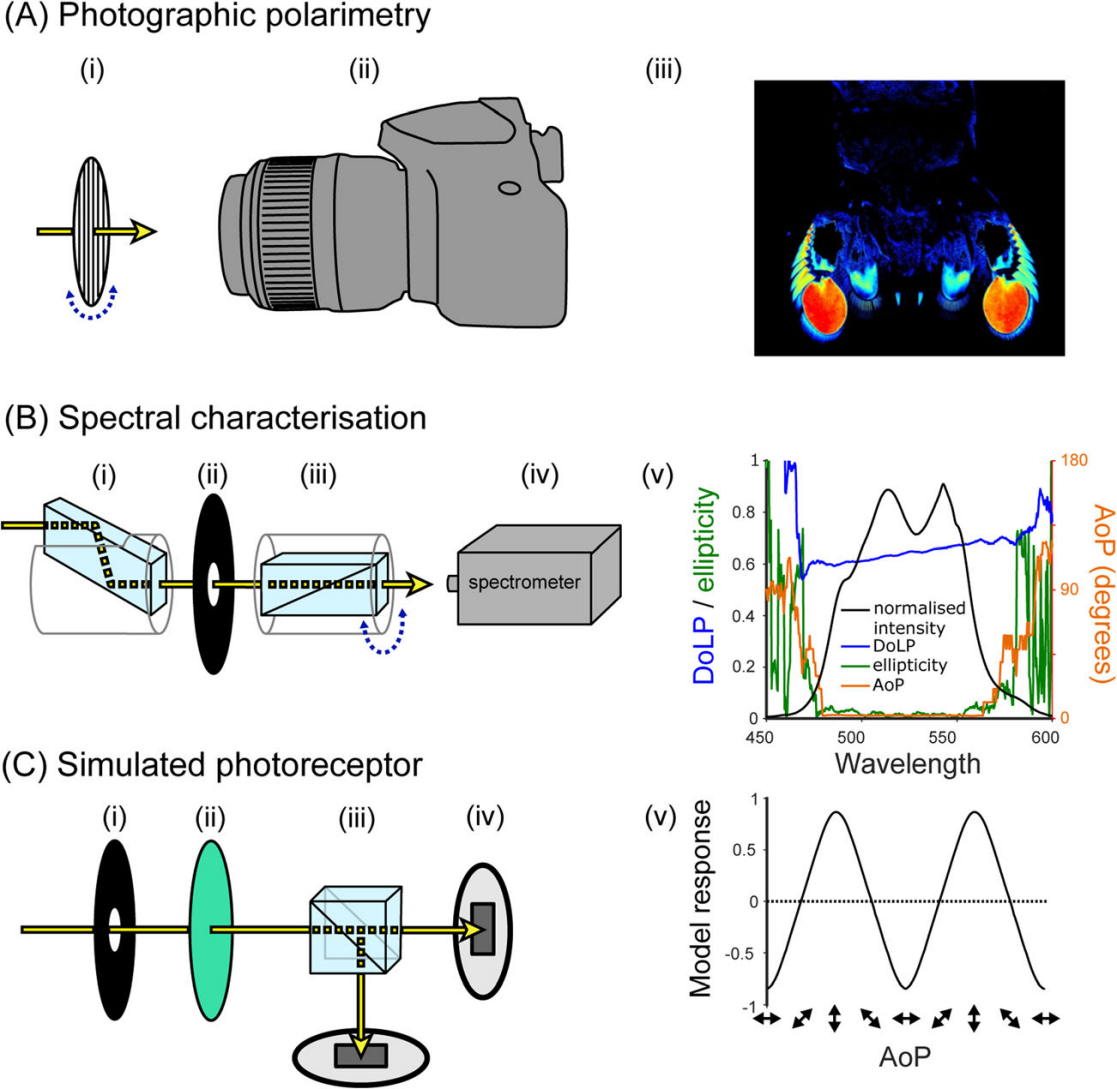
Example systems for measuring polarisation and the types of data they produce. **a** Photographic polarimetry using (i) a rotatable linear polarisation filter and (ii) a camera in full manual mode. False colour images (iii) can be used to represent the spatial distribution of polarisation. **b** Spectral characterisation of polarisation using (**i**) a Fresnel rhomb, (ii) an aperture, (iii) a rotatable Glan-Thompson polarizer, and (iv) a spectrometer. Polarisation characteristics may then be plotted against wavelength (v). Note: Stokes parameter calculations are only accurate where the intensity is sufficiently high (here: 470–570 nm). The noise at < 470 nm and > 570 nm is shown to illustrate the erroneous information resulting from insufficient intensity. **c** Simulated photoreceptors using (i) an aperture, (ii) an interference colour filter, (iii) a polarisation filter (in this case a polarising beam splitter), and (iv) photodiodes. Output can be simplified into a single measure of contrast between the two detectors (“model response”) (v)

The use of photographic cameras for gathering polarisation information is one of the simplest and most widely implemented approaches. Because of its simplicity, it is also highly prone to misuse and misinterpretation, so an understanding of the benefits and pitfalls of this technique is essential. At its most basic level, all that is required for photographic polarimetry is a standard photographic polarisation filter (Fig. 4a, i), marked around its perimeter for the desired set of polarizer orientations, and a stable photographic camera with the capacity for full manual control of focus, aperture, exposure and subsequent image processing (Fig. 4a, ii). Photographic linear polarisation filters and so-called circular polarisation (CP) filters are both perfectly adequate for this task, as long as they are mounted on the camera in the standard way. Note that many modern cameras use partially-reflecting mirrors to control autofocus and light metering. These mirrors reflect polarisation differently depending on its angle, and incoming light that is highly linearly polarised (as is the case for a linearly polarising filter) can affect these automatic processes. The problem can be partially mitigated by using CP filters, and by manually controlling focus and exposure.

A typical protocol for photographic polarimetry would be to take a series of images of a scene through the polarisation filter angled at 0°, 45°, 90° and 135°, then process this set of images pixel-by-pixel to determine the differences in brightness between them (i.e. the component of the scene that is polarised). Converting image contrasts to polarisation information can be achieved in several ways (e.g. Wolff 1990; Schechner 2011; Foster et al. 2014), one of which is based on calculating the Stokes parameters.

Once the polarisation information has been calculated, there are a number of methods for presenting it in a visually intuitive manner. This typically involves the use of colour maps (but see recent work by Gagnon and Marshall 2016, for an alternative) to represent the main aspects of polarisation: DoP, AoP, and in some cases, ellipticity (e.g. Fig. 2). Colour maps may also reflect the contrast sensitivity of the animal, in terms of the threshold at which they can distinguish two different angles or degrees of polarisation. This can be achieved by adjusting the bit-depth of the colour map to provide information about what is actually visible to the animal (e.g. Temple et al. 2012). It should be noted that no animals are known to perceive the DoP and AoP as separate qualities of light (Labhart 2016), but it is nonetheless informative to consider spatial patterns of polarisation in this way. Some attempts have been made to combine different polarisation information within single images, for example by representing the AoP using hue of a colour map and the degree as saturation (Foster et al. 2014), while others have converted polarisation information into an estimate of photoreceptor activity in a biological system, which is then represented in false-colour images (How and Marshall 2014; How et al. 2015).

There are a number of constraints in the technique of photo-polarimetry. Firstly, the camera and the visual scene need to be as stable as possible throughout image acquisition. Edge effects and small movements of the camera between images necessitate an image registration step, and objects moving in the scene can result in false polarisation signatures. Secondly, the aperture and exposure controls of the camera must remain unchanged across the image series and be set so that the areas of interest in the scene are neither under nor over exposed. Such situations are often unavoidable in parts of the image (e.g. facing into the sun) but should be highlighted as falsely exposed in subsequent processing steps or dealt with using multiple exposures (exposure bracketing). Thirdly, and perhaps most importantly, dark areas of images are particularly vulnerable to producing erroneous polarisation signals. This is because, when comparing polarisation images in subsequent processing steps, the polarisation information is calculated from differences in small numbers (close to the zero end of the sensor scale) that have associated noise. Sensor noise can result in artificially inflated estimates of polarisation in these areas (Tibbs et al. 2018). We recommend excluding pixels with values in the lower 5% of the sensor range from analysis. Fourthly, care must be taken when using wide-angle lenses with frontally mounted polarisation filters. Towards the periphery of these images light passes through the polarisation filter at oblique angles, potentially inducing intensity artefacts (see Viewing-Angle Effects in Polarizers). Finally, photographic cameras rarely have a linear sensitivity to changes in light levels: i.e. contrasts in light levels are measured differently at the bright end of the camera sensor’s range to the dark end. The problem is especially pronounced for film cameras, for which responses to light intensity are non-linear throughout the functional range, but it is also somewhat true of the charge-coupled device (CCD) or complementary metal–oxide–semiconductor (CMOS) chips of digital cameras. This problem can be reduced by deactivating any digital adjustments made to the images by internal processes within the camera (such as auto-white-balance and gamma corrections), and using the raw image format where available (e.g. DCRAW: Coffin 1997). As a final step, the quantitative accuracy of photographic polarimetry measurements can be greatly improved by determining the sensitivity curve of the camera chip (Stevens et al. 2007; J. Smolka and D.-E. Nilsson, in preparation) and lens distortion (J. Smolka and D.-E. Nilsson, in preparation), and compensating for these to linearise the intensity scale.

While there are limitations to the accuracy of photo-polarimetry, it remains a useful technique for investigating the relative distribution of polarisation information across scenes. It is particularly useful for highlighting areas of interest in natural scenes (such as polarisation signals; see Biological Polarizers), and for discovering possible polarisation artefacts in experimental setups (e.g. Egri et al. 2016). The recent development of division of focal plane video camera CCD chips, with adjacent pixels etched with different polarisation filters may improve temporal, spatial and measuring accuracy in photo-polarimetry, by allowing for the continuous filming of polarisation in moving scenes (Powell and Gruev 2013; York et al. 2014). There have also been developments in engineering camera chips that are inherently sensitive to the polarisation of light (Park and Crozier 2015). Researchers interested in circular polarisation patterns can also augment photo-polarimetry using a frontally mounted quarter-wave retarder filter (e.g. Gagnon and Marshall 2016). Such filters are usually expensive and function only in a restricted part of the spectrum, so care should be taken to analyse only the appropriate colour channel of the camera or introduce a colour filter to restrict the spectral range.

#### Spectral characterisation

An effective lab-based system for measuring a single source of polarisation involves using a spectrometer coupled to a polarisation filter that functions across the light source’s spectrum (Fig. 4b). Spectrometers are the measurement device of choice among visual biologists. These devices split the measured light across a range of wavelengths using a diffraction grating, and the light is then detected with a CCD chip. Methods for calibrating and using spectrometers are beyond the scope of this review, however, their manufacturers often provide detailed online tutorials (see also: Johnsen 2012, chapter 9).

The optical properties of the spectrometer’s diffraction grating can induce measurement artefacts if incoming light is polarised. It is therefore important to ensure that the spectrometer receives depolarised light only. While this can be achieved by adding a diffusing component between the polarizer and the spectrometer, the simplest method is to use a long, looped multimode optical fibre, as internal stresses in the coiled optic fibre help depolarize the beam along the fibre’s length (Yu and Rawat 1992). Our measurements suggest that a minimum of 2 m of coiled fibre are required to eliminate this artefact (see supplement S2).

To measure the Stokes parameters, a spectrometer must collect light transmitted through a rotatable linear polarizer. Glan Thompson or Glan Taylor linear polarizers (Fig. 4b, iii) are often used, since these polarizers have high extinction ratios across the UV and visible spectrum. To measure the ellipticity of the incoming light, a Fresnel rhomb is added before the linear polarizer in the light path (Fig. 4b, i). These devices are intrinsically achromatic (supplement S5), a property that is essential for the characterisation of polarisation across a broad range of wavelengths (see supplement S1 for an example setup).

The procedure for measuring Stokes parameters is similar to that used for photo-polarimetry. A series of spectrometer readings is stored for a set of polarizer angles (typically 0°, 45°, 90° and 135°), and then the Stokes parameters S_0_–S_2_ can be calculated for each wavelength (see also: Wolff 1997). To measure ellipticity, the Fresnel rhomb is added to the light path and two further measurements taken with the linear polarizer rotated − 45° and + 45° relative to the axes of the Fresnel rhomb.

There are several details to bear in mind when building and using such a device. Firstly, it is important to make sure that the light collected by the spectrometer consists exclusively of light that has passed through the polarisation filter(s). The linear polarizer and Fresnel rhomb often come mounted in aluminium holders that do not exclude the possibility of light leaking around its edges. This can easily be remedied by introducing an aperture that restricts the range of angles at which light enters the polarizers (Fig. 4b, ii). Secondly, as for the previous example, the measurement apparatus and the light environment need to remain stable during measurement collection. Changes to any of these could create differences between the measurements. Finally, the Fresnel rhomb displaces the optical path of the sampled light (Fig. 4b, i), so this needs to be compensated for, especially when measuring small objects.

#### Simulated photoreceptors

In situations where the visual system of the study animal is well characterised, measurements can be made to reflect its sensory responses to polarised light more closely. The following example uses pairs of photodiodes to simulate an animal’s polarisation-sensitive photoreceptors (Fig. 4c, iv). It is important to note the sensitivity, gain, and dark current levels for photodiodes, particularly when comparing input from multiple channels, so a calibration step is likely to be necessary (see Lambrinos et al. 1997, for an example). The measured light beam is filtered by a non-polarising colour filter, such as a glass interference filter (Fig. 4c, ii), to approximate the spectral sensitivity of the animal’s photoreceptor or to restrict the analysis to a specific set of wavelengths. Polarisation sensitivity is conveyed by the addition of linear polarizers at angles that match the angular sensitivity of cells in the animal’s visual system (to compare 0° and 90°, a beam splitter might suffice: Fig. 4c, iii). As for spectral characterisation, the acceptance angle of each photodiode should be controlled via an aperture (Fig. 4c, i). Because the extinction ratios of linear polarizers are far greater than the most effective detectors found in nature, the signal from the photodiode needs to be transformed to better approximate the animal’s polarisation sensitivity.

Throughout this manuscript, we refer to situations in which measurements of the stimuli and experimental arena are required. Measurements of polarisation, radiance and the illumination spectrum help to ensure that the animal observes a stimulus with the intended properties (below) and is prevented from viewing alternative cues that may confound a study’s conclusions (see Confounding Cues in Experiments).

## Producing polarised stimuli

Just as there is a variety of different sources of polarised light in nature, a range of methods can be used for producing and controlling the polarisation of light under laboratory conditions. Although many discoveries have been made in the field of animal polarisation sensitivity using nothing more than a sheet of polarizer and an appropriate light source, in recent years a number of more flexible and sophisticated methods have been developed that permit the production of more complex or naturalistic stimuli. Although these methods place the experimenter in control of the polarisation of the visual stimuli, they are not necessarily straightforward or entirely predictable. Where polarised stimuli are used the only sure way to know if their properties meet the requirements of the experiment is to measure them, preferably in situ, using one of the methods we have outlined above.

### Manipulating angle of polarisation

While all methods for producing polarised light can be used to manipulate degree of polarisation to some extent, the aim, in most cases, is deterministic control of angle of polarisation. By far the most common method for doing this is to place a sheet of polarising filter between the light source and the observer. In some instances, specular reflections from smooth surfaces have also been used to mimic those from bodies of water. More novel methods, such as the modified LCD monitors and “scattering tanks” described below, may permit the production of patterns in angle of polarisation, adding spatial detail to polarised stimuli.

#### Sheet polarizers

Polarising films, mounted between sheets of plastic or glass, are the most commonly used polarizers in biological studies. Transmitted light is polarised via maximal absorption of a specific angle of polarisation coinciding with the direction of the long-axis of absorptive molecules in the film (Land 1951). The AoP perpendicular to this axis, which is absorbed minimally (and hence transmitted maximally), is termed the polarizer’s *transmission axis* (TA). By rotating the polarizer, stimuli with any AoP can be produced. For this type of polarizer, extinction ratios typically range between 10^−3^–10^−4^. Two main disadvantages of sheet polarizers are spectral transmission and changes in intensity with off-axis transmission (see Confounding Cues in Experiments). Manufacturers typically state the effectiveness of sheet polarizers across the visible range and, although some plastic mounted polarizers perform poorly in the UV, more expensive ones can perform better (supplement S3). Indeed, most studies using sheet polarizers have chosen models, such as Polaroid’s (now out of production) herapathite-neutral ’B (HNP’B), that transmit and polarise well across the UV–visible spectrum (see supplement S3).

#### Specular reflections

Flat surfaces partially polarise light upon reflection, so the production of polarised reflections requires no specialised equipment. In a series of studies, Horváth and colleagues made use of thin sheets of black polyethylene (review: Horváth and Kriska 2008) to produce strongly polarised reflections. Panes of glass (Schwind 1983, 1989, 1991, 1995; Shashar et al. 2005) and oil filled trays (Horváth and Zeil 1996; Egri et al. 2012) have proved similarly effective. Because these techniques do not require materials that are either expensive or delicate, they are easily applied in field studies, and have led to the identification of more than 250 species of insect as potentially polarotactic (review: Horváth and Kriska 2008).

**Fig. 5:**
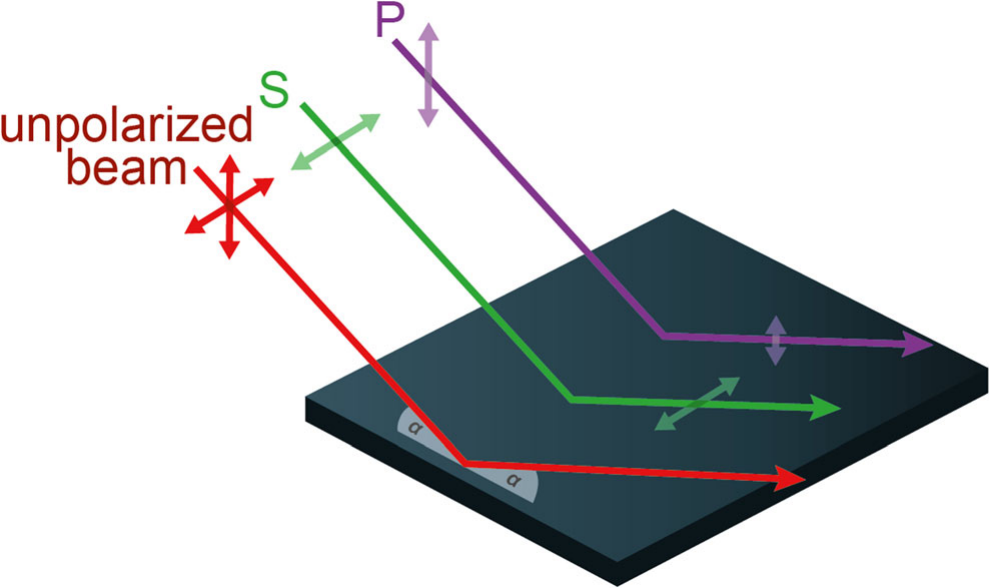
Specular reflection of S- and P-polarisation. An unpolarised beam (red) that is specularly reflected from a smooth surface can be considered as a combination of two linearly polarised components. One of these is polarised parallel to the surface (s-polarised: green) and preserves more of its intensity on reflection (green double-headed arrow length) than the component polarised perpendicular to it (purple double-arrow length). The angle *α* illustrates that the angle of incidence is equal to the angle of reflection (grey shaded angle)

At a non-metallic surface, a greater proportion of the intensity component that is polarised parallel to a surface (s-polarised) is reflected compared with the component polarised perpendicular to that (p-polarised; i.e. polarised parallel to the plane of incidence, the plane containing the incident and reflected rays). As a result, reflected light is polarised parallel to the reflective surface (Fig. 5). The DoP of reflected light varies as a function of the angle of incidence, reaching a maximum at Brewster’s angle, *θ*_***B***_ measured from the reflecting surface, (Brewster 1815) with

9 $$ {\theta}_{\boldsymbol{B}}=\arctan \left(\frac{n_1}{n_2}\right) $$

where *n*_*1*_ and *n*_*2*_ are the refractive indices of the initial medium and the reflecting medium respectively. This function limits the range of viewing angles at which high-DoP reflections occur. Since refractive index also varies as a function of wavelength, a polarised stimulus created in this fashion can vary in DoP as a function of wavelength.

When reflections are used as a source of polarised light, the spectral and viewing-angle effects require appropriate controls. Control stimuli are typically surfaces with high reflectance averaged across viewing angles, this being a quantity that takes into account contributions from both specular (mirror-like) and non-specular (diffuse) reflection from a surface, including sub-surface scattering and surface reflections from microfacets not aligned with the dominant surface plane (Wolff 1990). Hence, these surfaces are typically lighter coloured equivalents of the same material (e.g. Schwind 1983, 1989, 1991; Kriska et al. 1998; Kriska et al. 2006; Csabai et al. 2006; Horváth et al. 2007; Kriska et al. 2009; Boda et al. 2014). While reflections from these surfaces are lower in DoP, measurements are required to ensure that other cues are not introduced by the change in intensity and reflectance spectrum. For this reason, we repeat our earlier recommendation that polarising filters may be used as an alternative to true surface reflections, where it is specifically the detection of polarisation that is of interest.

#### Twisted nematic (TN) liquid crystal displays

While polarising filters are useful for modifying light in a uniform and stable manner, some researchers have turned to liquid crystal display (LCD) technology to produce complex and dynamic polarised stimuli (e.g. Glantz and Schroeter 2006; Temple et al. 2012; How et al. 2012; Daly et al. 2016). These displays, which currently include many flat-panel computer monitors, digital projectors and televisions, generate brightness and colour contrasts by manipulating the polarisation of light.

**Fig. 6:**
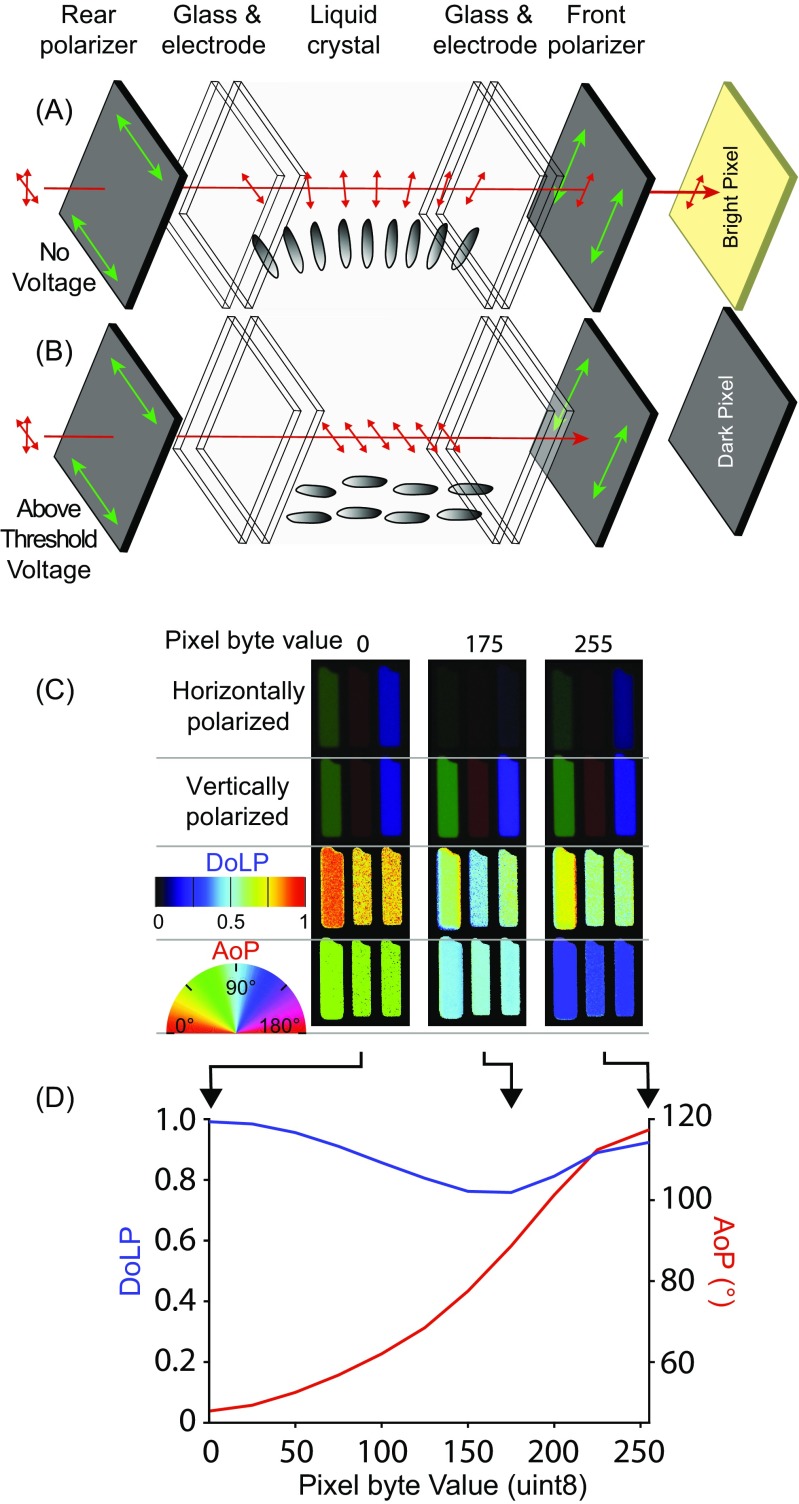
A twisted nematic (TN) panel that can be converted to an angle-of-polarisation monitor. **a** Schematic of TN LCD Monitor Layers. Illumination (red) from the monitor’s light source first passes through the rear polarizer, for which the transmission axis (green arrows) is perpendicular to the front polarizer. The long axes of the liquid crystal molecules (grey ellipses) twist through 90°, causing a 90° rotation of the AoP. As a result, the light is transmitted through the front polarizer. **b** When a voltage is applied, above a threshold, the molecules start to reorient, becoming perpendicular to the glass substrates. At a sufficiently high voltage, the 90° twist is completely removed, and the liquid crystal layer no longer rotates the AoP. As a result, the light is absorbed by the front polarizer. A change between 0° and 90°, via application of an intermediate voltage level, results in a pixel of intermediate brightness. **c** Imaging polarimetry (see How Polarised Light is Measured) showing red, green and blue sub-pixels in a TN panel (Dell 1908FPC) converted to an angle-of-polarisation monitor via removal of the front polarizer. Pixel byte values for 0 (black), 175 (grey) and 255 (white) shown, each producing a different AoP. Each pixel is 293 μm tall and wide (i.e. across the red, green and blue sub-pixels). **d** AoP and DoLP characterisation of the monitor’s output across the 256 interval input scale, showing the gradual change in rotation of the AoP of light as the input value increases (decreases in voltage for a TN panel)

LCDs consist of a liquid crystal panel with electrodes sandwiched between two linear polarisation filters oriented at 90° to one another (Fig. 6 a–b). Twisted nematic (TN) LCDs can be used to control the AoP of transmitted light. In a TN device the alignment layers are deposited at 90° to each other on the inside of glass of the LCD panel. This induces a 90° twist in the long axes of the liquid crystal molecules (Fig. 6a) through the device. This quarter turn of a helix has the appropriate properties to guide the AoP, such that AoP rotates through 90° and the outgoing light is aligned with the transmission axis of the front-most polarizer. This creates a bright pixel. When a voltage above a specific threshold is applied, the liquid crystal molecules reorient, with their long axes becoming parallel to the light path (Fig. 6b). In this case, light passes through the liquid crystal with its AoP unaltered and is absorbed by the front polarizer. This creates a dark pixel. The addition of red, green and blue coloured filters in neighbouring pixels allows the full control of colour that we are familiar with on our computer monitors.

Converting a TN LCD monitor from a colour-and-brightness monitor to a polarisation monitor is a simple process and involves only removing the front-most polarizer. The result is that transmitted light is uniform in intensity, but varies in polarisation. The exact polarisation properties of the transmitted light across the 256 levels of voltage applied to each pixel varies according to (1) the colour channel; (2) the inbuilt brightness and contrast settings of the monitor; and (3) the brand and type of LCD panel. There may also be measurable variations within models caused by differences in components. Digital projectors that use a liquid crystal device can also be modified in a similar way. However, the outermost polarizer film can be difficult to access and is sometimes located on three different LCD components, one for each colour channel. Patterned vertical alignment (PVA) LCD monitors can also be used to present polarised stimuli (Fig. 8), permitting control over degree- rather than angle of polarisation (see below).

A number of further modifications can be useful for adapting LCD monitors for polarisation experiments. Replacing the inbuilt light source with an alternative, purpose-built source can be beneficial for altering the brightness or spectrum of the output light. Opening the rear of the LCD panel to replace the light source usually requires the removal of the power source and control circuitry (which must then be housed separately). Removing unwanted colour channels helps to reduce the spectral complexity of the transmitted light stimulus, simplifying the interpretation of behavioural results. This can be achieved by placing appropriate colour filters between the light source and the rear-most surface of the LCD panel (many colour filters affect polarisation and should therefore be placed before the rear polarizer in the light path). Finally, the range of angles of polarisation produced by LCD monitors tends to be around 90°, but can be shifted, relative to real-world coordinates, by mounting the monitor on a rotatable stand. For example, a monitor for which AoP can be adjusted between − 45° and 45° to vertical, can be rotated by 45° to produce a stimulus that can be varied between 0° and 90°.

The most important tasks throughout all modifications and adjustments are the measurement and calibration of the monitor’s output (see supplement S8 for more detail). Accurate measurements are critical to determine the range of stimuli a monitor produces. LCD monitors seldom produce linear changes in polarisation (Fig. 6d). For example, for displays with an AoP ranging between − 45° and 45°, the change in angle relative to the byte value addressed to each pixel is non-linear and is usually accompanied by changes in ellipticity (and therefore DoLP), in some monitors reaching as high as 60% for intermediate pixel values.

One concern when using LCD monitors for polarisation experiments is that they may produce intensity artefacts for oblique viewing angles. Just like polarising filters (see Viewing-Angle Effects in Polarizers), LCDs function optimally for light incident perpendicular to the monitor’s surface (see supplement S7) and we therefore recommend that the experimental animal is positioned to avoid oblique viewing angles (see Controlling for Intensity Confounds). In cases where this cannot be achieved, these potential intensity cues should be measured and taken into consideration.

#### Manipulating the angle of polarisation using scattering

**Fig. 7:**
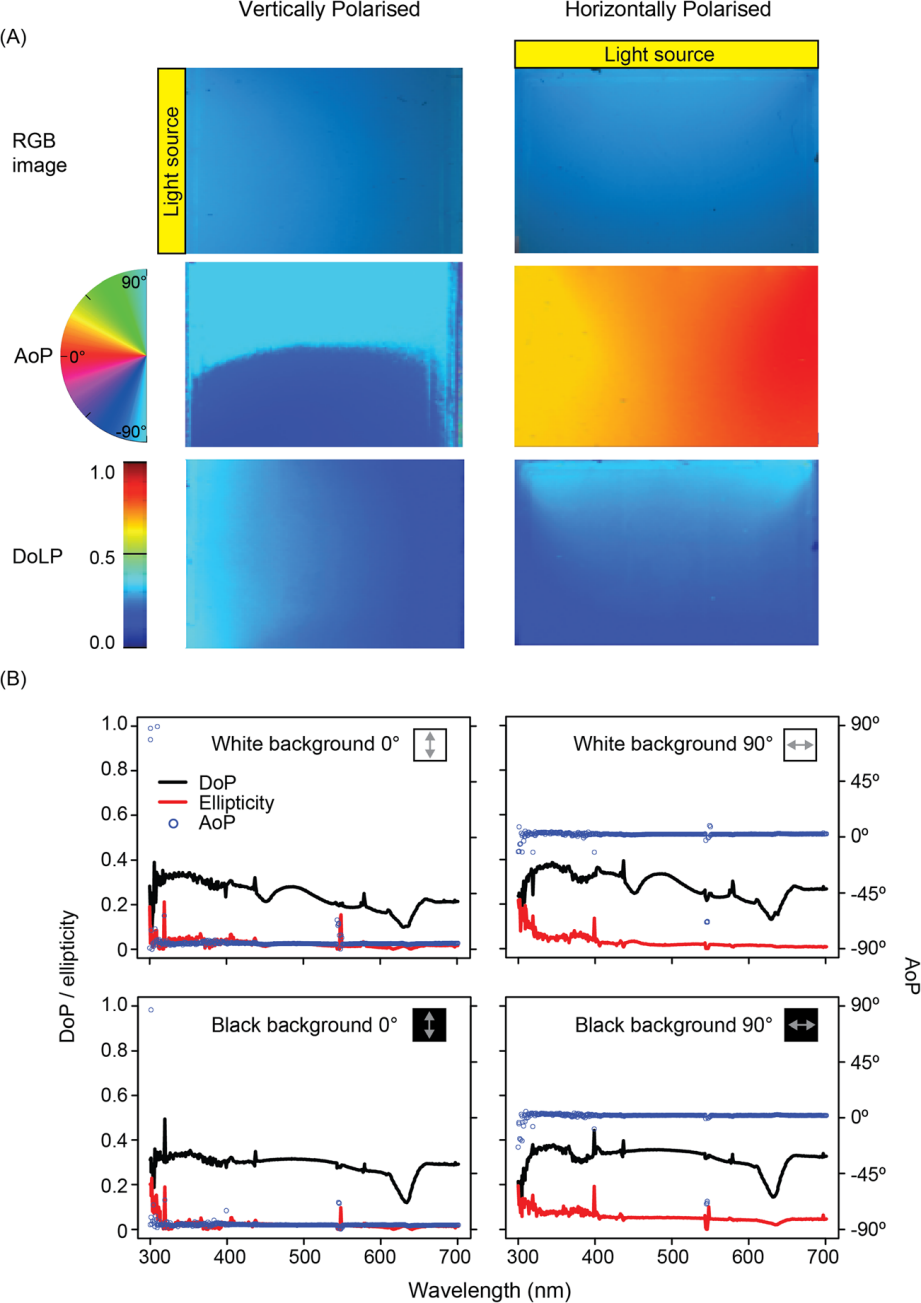
Scattering tank used to manipulate AoP. **a** True-colour and false-colour (polarisation) images of a scattering tank oriented to produce either vertically or horizontally polarised light. Light source and polarizer either to the side of the tank (left) or on top of the tank (right) producing vertically and horizontally polarised scatter, respectively. Colour images (top), photo-polarimetric images of angle of polarisation (middle), and degree of linear polarisation (bottom). **b** Spectral polarisation measurements, with a monitor displaying either white (top) or black (bottom) transmitted behind the tank. *N.B.* At the monitor’s emission peaks (e.g. 450 nm, 550 nm) the white unpolarised background reduces the observed DoP

Just as scattering is a common source of polarised light in nature, under laboratory conditions both the angle and degree (below) of polarisation can be controlled by using suspended particles as a scattering medium and manipulating the illumination (Fig. 7). This method creates a broad-field polarised stimulus, and can be used to mimic veiling light, by combining unpolarised light (transmitted straight through the medium to the observer) with scattered polarisation (scattered at 90° towards the observer). An example of a scattering medium might be a mixture of water and sub-wavelength sized particles, such as those found in skimmed milk (Sharkey et al. 2015), contained within a glass or clear acrylic tank. When the light illuminating the tank from above is unpolarised, a proportion of that light undergoes Rayleigh scattering, creating a weakly polarised light field. If the illumination itself is polarised (e.g. by introducing a polarizer before the tank), it follows that a greater proportion of the side-scattered light is polarised.

The AoP of this scattered light can be controlled via the illumination. Since side-scattered light is polarised perpendicular to both its original and final direction of travel (see Sources of Polarised Light in Nature), the whole arrangement can be rotated around the study animal’s viewing direction, to produce a light field in which AoP is also rotated with the direction of illumination (Fig. 7). Rotating the AoP of the illumination by 90° also results in a shift in the predominant AoP of the light scattered within the tank by 90° for the original viewing direction. It should be noted that this also reduces the intensity of the scattered light in this direction, which can be compensated for by brightening the illumination. This also reduces the degree of polarisation of light scattered in the original direction, which cannot be easily compensated for, but has applications in itself (see below). These techniques are intended to mimic scattered light in an aquatic environment, which may conceal objects (Lythgoe and Hemmings 1967) or form a polarised background from which objects must be distinguished (Johnsen et al. 2011). One application is to test the advantages of polarisation sensitivity in contrast sensitivity through veiling light, using a naturalistic polarised light field (Sharkey et al. 2015).

### Manipulating degree of polarisation

Most experiments testing animal polarisation sensitivity to date have used polarising filters that produce polarised light with a DoP approaching 100% but, under natural conditions the degree of polarisation is often lower: celestial polarisation patterns rarely exceed 85% (Cronin et al. 2006; Horváth et al. 2014) and underwater polarisation typically only exists to a maximum of 50% (Lerner 2014). To assess an animal’s response to polarised light under more natural conditions, stimuli should be produced with DoP values comparable to those in its natural visual environment. Such stimuli also make it possible to characterise the limits of the degree of polarisation that an animal can detect, i.e. measure the DoP threshold (Labhart 1996; Glantz and Schroeter 2006). Methods for controlling the DoP fall into four categories: scattering/diffusion, adding unpolarised light, modified LCDs, and retardation devices.

#### Manipulating degree of polarisation using scattering

Scattering can be a source of polarised light, but it can also decrease the DoP (e.g. clouds). The difference is determined by the type of scattering, which depends on the size of the scattering particles and their density. If the particles are very small (i.e. less than λ/15, where λ is the wavelength of light) then Rayleigh scattering occurs. If the particles are larger (i.e. approaching the size of λ) then Mie scattering occurs (Mie 1908). Mie scattering and multiple Rayleigh scattering events can be used to alter the effective DoP of a transmitted beam. In the laboratory, suitable methods to scatter light involve particulates (e.g. sand or hollow glass spheres) suspended in liquids (see above) and translucent materials.

A simple scattering filter can be constructed by using the scattering tank method described above, and keeping it well mixed. The DoP can then be altered by varying the concentration of scatterers or the path length of light passing through the mixture or the size of the particles. Key considerations are that the tank and scatterers do not vary the spectral characteristics between different DoP conditions and that the tank does not vary the ellipticity of the light. If the specific density of the particulate differs from that of the liquid, it may settle at corners and edges of the tank and it is therefore important to measure the system after it has been set-up and mixed for some time.

Translucent materials offer the easiest means of decreasing DoP, and commercially available diffusion filters can be obtained from photographic and theatrical suppliers, as well as makers of optical components. These are glass, polymer or thin film (gel) diffusers with varying levels of scattering ability. They are not sold as DoP filters nor do they come with any quantification of how they affect DoP, but are nonetheless effective. High-end diffusers may provide a point spread function, describing the distribution of diffused light, and lower-end diffusers may give an arbitrary value that indicates the extent of scattering. The interaction of diffusers with polarisation depends, in part, on the distance of the diffuser from other components in the light path: a diffuser positioned close to a polarizer has less of an effect on DoP than one further away.

Among high-end diffusers, frosted quartz best transmits UV, with frosted glass or plastic diffusers only effective at depolarizing light between 400 and 800 nm. In addition, glass and quartz diffusers do not induce elliptical polarisation; as opposed to frosted plastics, which can induce ellipticity when heat stressed. However, glass and quartz can be prohibitively expensive, especially for large filters. Alternatively, purpose-made frosted diffusers can be produced via sandblasting or acid etching. Numerous filters can also be stacked to decrease the DoP (e.g. Egri et al. 2016), but note that the effects of multiple filters are not, in general, linearly additive, and so each condition produced this way must be measured separately. Frosted film (polyester/gel) diffusion filters are available in a wide range of densities, as are grid cloths and spun materials. These are cheaper than frosted glass, plastic or quartz and may be preferable for larger stimuli. One important caveat when using thin film diffusers to control polarisation is that these films can act as retarders. As a consequence, the orientation of the diffusers must be controlled and maintained relative to the polarizer to control the DoP consistently.

Various researchers have used scattering to decrease the DoP in their experiments. Hawryshyn and Bolger (1990) used latex microbeads of 1 μm diameter to decrease the polarisation of stimulus light. Schwind (1999) used milk-and-water scattering tank, similar to the one described in the previous section, in which the DoP of stimulus light decreased as a function of increasing distance travelled by that light through the scattering tank, between the polarizer and the observer. Henze and Labhart (2007) used 2 sheets of translucent tracing paper on a sheet of polarizer to reduce DoP to zero, reversing the order to get a DoP of one. Cartron et al. (2013) used “fine natural sand” of < 1 mm diameter to decrease DoP and intensity contrast of images displayed on a modified LCD and CRT monitor respectively. Temple et al. (2015) used “custom-made volume diffusers” to decrease the DoP of stimuli, based on the same principle of adding particulates to a transparent medium. In each case, since the process of depolarisation in a scattering medium is not easy to predict, the measurements of degree of polarisation were reported alongside estimates of the DoP or target detection thresholds, as evidence that the scattering media reduced the DoP of stimulus light.

#### Adding unpolarised light

Another method to decrease the DoP of an already polarised light source or field is to add unpolarised light into the optical path, reducing the proportion of light that is polarised when it reaches the viewer. A simple mechanism for doing this is to position a sheet of glass at an angle between the viewer and the polarised light field. An unpolarised light field can then be reflected from the glass into the optical path, and by adjusting the relative intensity of the polarised and unpolarised light fields, a range of DoP values can be attained. A drawback of this system is that the viewing angle for which it is effective is limited. While the authors have used this approach in a preliminary experiment, we know of no published work that has employed the beam splitting approach in animal behavioural studies.

#### Patterned vertical alignment (PVA) liquid crystal displays

**Fig. 8:**
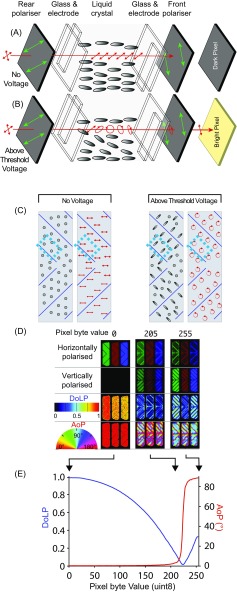
A patterned vertical alignment (PVA) panel that can be modified to act as a degree-of-polarisation monitor. **a** Schematic of PVA panel layers. The transmission axes of the rear and front polarizer are perpendicular, and a dark pixel is achieved by allowing the illumination (red) to pass through the liquid crystal unmodified. The indium tin oxide electrodes are depicted by the smaller rectangle inside the glass. **b** For different applied voltages, the liquid crystal layer retards output light to different extents, altering its DoLP. **c** Front view of a pixel, showing the liquid crystal alignment (grey ellipses) and polarisation state of output light (red arrows). The blue boxes indicate the area of the pixel represented in (**a**) and (**b**). The two diagrams show the cases of no voltage (left) and an above threshold voltage (right). For an above threshold voltage, linearly polarised light is converted to equal quantities of left and right-handed elliptically polarised light in the different domains of the chevron, which combine to cancel out, and change the DoP of the output light. At a particular voltage, the domains act as quarter-wave retarders, changing the polarisation into left and right-handed circular polarisation, which cancel to give a DoP of zero. **d** A modified DoLP monitor (Dell 1905FP) at the pixel level. Polarisation changes in PVA displays are complex, varying within domains in each pixel. Conversion of horizontally to vertically polarised light begins at intermediate byte values (e.g. 205), in the regions of the pixel domains in which the liquid crystal molecules tilt. In (**a**) to (**d**) the width of the electrodes is approximately 100 μm, and each pixel is 293 μm tall and wide. **e** Across most of the input range, the averaged AoP of the monitor’s output remains the same while DoLP decreases with increments in pixel byte value (i.e. increases in voltage for a PVA panel)

As described earlier, LCD monitors can be modified to present dynamic or static polarisation contrasts instead of intensity and colour contrasts. While models of twisted nematic (TN) LCDs can be modified to produce changes in the AoP across the screen (Glantz and Schroeter 2006; How et al. 2012; Temple et al. 2012; Daly et al. 2016), other LCD technologies can also change the DoP. The differences between LCDs that produce changes in the AoP versus the DoP relate to the alignment of the liquid crystal. In patterned vertical alignment (PVA) LCDs, the liquid crystal molecules are oriented parallel to the light path (a homeotropic orientation) in the default state when no voltage is applied (Fig. 8a–c). As a result, the polarisation of transmitted light is not modified by the liquid crystal, producing a dark pixel.

In a PVA monitor, the electrodes are structured such that they are laterally offset between the two substrates. In addition, the electrodes are also shaped into chevrons, which together with the offset, creates four different domains per pixel when a voltage is applied. The molecules reorientate into the plane of the device at angles of 45°, 135°, 225° and 315° relative to the rear and front polarizers transmission axes. The change in the liquid crystal orientation causes a retardation effect, with different applied voltages creating different ellipticities. However, the symmetry of the four elliptical domains means the transmitted light is always comprised of equal quantities of left-handed and right-handed polarisation. These cancel each other out, resulting in a changing DoP. The PVA monitor that has been used in all experiments to date, (1905FP, Dell, Round Rock, USA) can vary the DoP from 0 to 1, which allows for testing of the minimum difference in DoP between a stimulus and its background that an animal can detect (How et al. 2014a). The monitor can also be rotated, to determine if this threshold changes at different orientations relative to the animal’s eye (How et al. 2014a).

#### Retarders

If the study animal is not differentially sensitive to the ellipticity of polarised light, a stimulus’ DoLP can be adjusted by increasing its ellipticity. When the AoP of the linearly polarised light beam entering a quarter-wave retarder is at 45° to the fast and slow axes of the retarder, it converts linear polarisation (ellipticity = 0, DoP = 1, DoLP = 1) into circular polarisation (ellipticity = 1, DoP = 1, DoLP = 0). If the AoP of the input light is aligned with either the fast or slow axis of the retarder, then there is no effect on polarisation and it remains linearly polarised (ellipticity = 0, DoP = 1, DoLP = 1). If the AoP of the input light is at any other angle to the fast or slow axis, the light is transmitted as elliptically polarised light (i.e. 0 < ellipticity < 1, DoP = 1, 1 > DoLP > 0). It should be noted that the value of retardation is not just a function of a material’s optical properties and thickness alone, it is also a function of wavelength (see supplement S4). Some materials act as retarders even though this is not their primary function, for example thin film diffusers and coloured theatrical gels made out of thin polyester or polycarbonate. When using such coloured and scattering filters, care should be taken to place them between the light source and polarizer (rather than between polarizer and animal) and measure any effects of orientation on polarisation.

Retarders have been frequently used for decreasing DoLP in behavioural experiments. Henze and Labhart (2007) used overhead transparency film and rotated the fast axis relative to the polarizer to vary DoLP from 1.0 to 0.0 in their experiments with field crickets. How et al. (2015) recently used a polymer film quarter-wave retarder (#88-252, Edmund Optics) when investigating the use of polarisation contrast by fiddler crabs. Other retarders used include drafting film (Mylar Medium Weight 0.003, Graffix: Tuthill and Johnsen 2006), photocopier transparency (Pfeiffer et al. 2011), polycarbonate gel filter (clear #00, Roscolux: Shashar et al. 2000) and laboratory film (Parafilm®, Bemis Flexible Packaging: Glantz and Schroeter 2006).

One of the main caveats in using retarders is that most (including optical-grade zero-order and multi-order retarders) alter the polarisation as a function of wavelength, and are often effective over a fairly narrow spectral range (see supplement S4). Thus, the wavelength of stimulus light should be restricted, or the spectral sensitivity of the polarisation photoreceptors must be known to fall within the desired range. As for all other methods for the production of polarised stimuli, careful measurement with reference to any information available on the animal’s visual system is vital for control over experimental conditions.

#### Projected polarisation

While this review was being compiled, a new method was published for controlling both angle and degree of linear polarisation in projected polarised stimuli (Stewart et al. 2017). Taking inspiration from a study in which light from a TN LCD was projected onto a translucent screen, producing moving stimuli that contrasted in AoP (Glantz and Schroeter 2006), the authors modified a digital light processing (DLP) projector to project moving patterns of polarised light (Stewart et al. 2017). While the LCD-based system produced stimuli with one DoLP level at a given time (using a laboratory-film retarder), the DLP-based system produces projected dynamic patterns that can be varied in AoP, DoLP, intensity and even wavelength (although this full range was not employed experimentally).

## Confounding cues in experiments

In experiments with polarised stimuli, it is important to control for confounding sources of stimulation. Since many of the optical effects that are responsible for the polarisation of light also affect its intensity and spectrum, it is always necessary to control for such changes where they may co-occur. These effects can be subtle and in cases where stimuli include UV light these confounds may not be detectable to the human eye. Failure to control for confounding cues can allow an animal’s response to be interpreted as polarisation sensitivity incorrectly or, conversely, can distract from true polarisation cues. In this section, we provide details of some potential sources of confounding cues in polarised stimuli and describe some methods to control for them.

### Sources of cue/confounds

#### Surface reflections

**Fig. 9:**
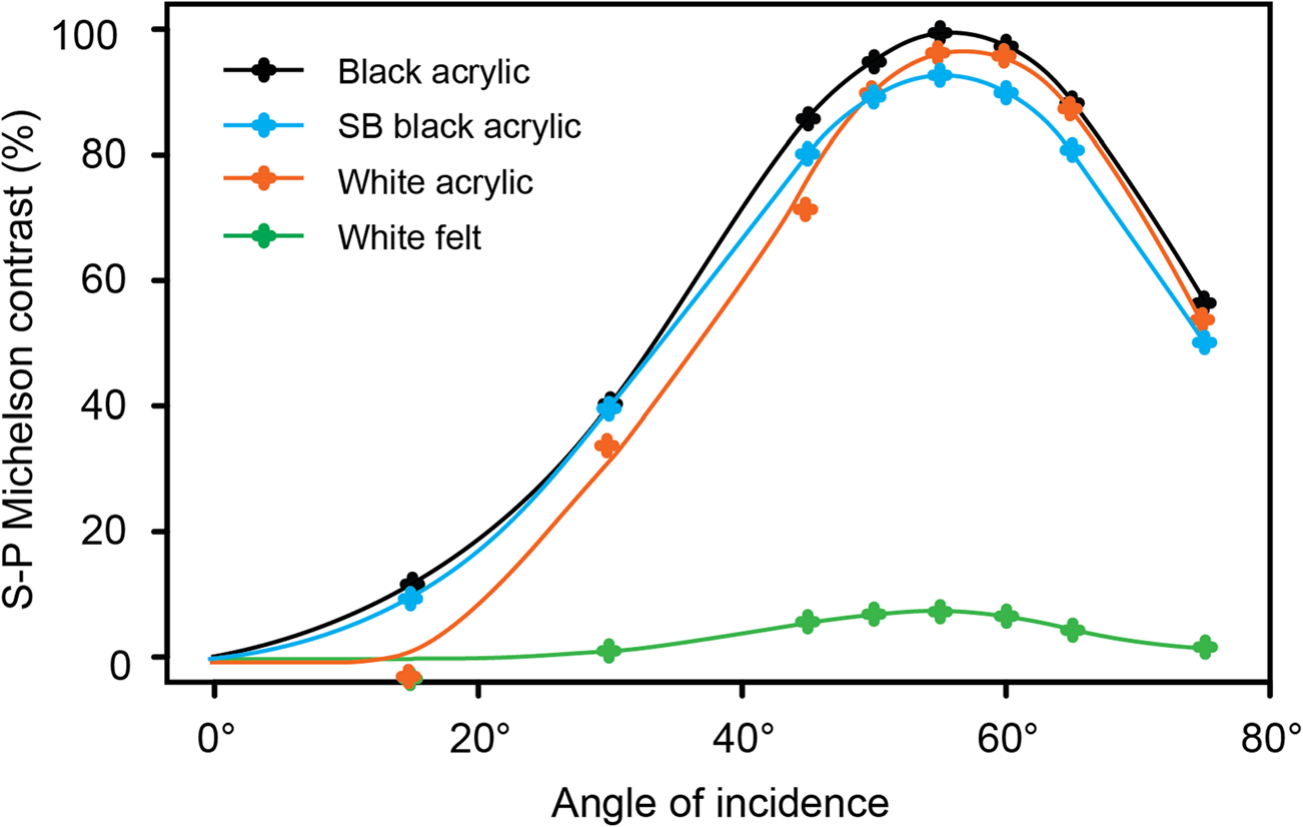
Contrast between reflected p- and s-polarised light. (Black) black acrylic (Perspex, Weybridge, UK). For angles of incidence 45–60° almost none of the p-polarised beam’s intensity is reflected light. (Orange) white acrylic (Perspex, UK). The difference in reflected intensity between s-polarised and p-polarised light is lower than for black acrylic. (Blue) the same block of black acrylic as above, sand-blasted to create a ‘rough’ surface. The increase in contrast with angle of incidence is more gradual than for untreated black acrylic. (Green) white felt (Fabric Land Ltd., UK). This material is both highly reflective and ‘rough’ (fibrous), and hence the contrast between reflected s-polarisation and p-polarisation is low at all angles. Raw measurements of reflected intensity available in the supplement (S5)

Specular reflections are a common and recurring issue in experiments with polarised stimuli (discussed in: Taylor and Adler 1973; Coemans et al. 1990; Goddard and Forward 1991; Horváth and Varjú 2004; Muheim 2011). Because a greater proportion of light that is polarised parallel to a surface (s-polarised) is reflected, compared with light that is polarised in the plane of incidence (p-polarised), the reflected intensity of a linearly polarised beam changes as a function of the AoP (Fresnel 1823). This causes the surface to appear lighter or darker depending on the AoP of the illumination. Light that is not specularly reflected or absorbed by a surface contributes to its diffuse reflectance (see Producing Polarised Stimuli—Specular Reflections), which affects a surface’s general lightness of appearance. While dark surfaces with low diffuse reflectances have been used to minimise the intensity of reflections in a number of studies of polarisation sensitivity (Rossel et al. 1978; Mussi et al. 2005; Sakura et al. 2012), this decrease in average reflectance increases the contrast between reflected s-polarised and p-polarised beams (Fig. 9), making the stimulus’ AoP discriminable via the brightness of specular reflections. This occurs because a smaller proportion of the beam’s intensity is reflected diffusely, irrespective of the AoP, but the same proportion is reflected specularly, as a function of the AoP.

**Fig. 10:**
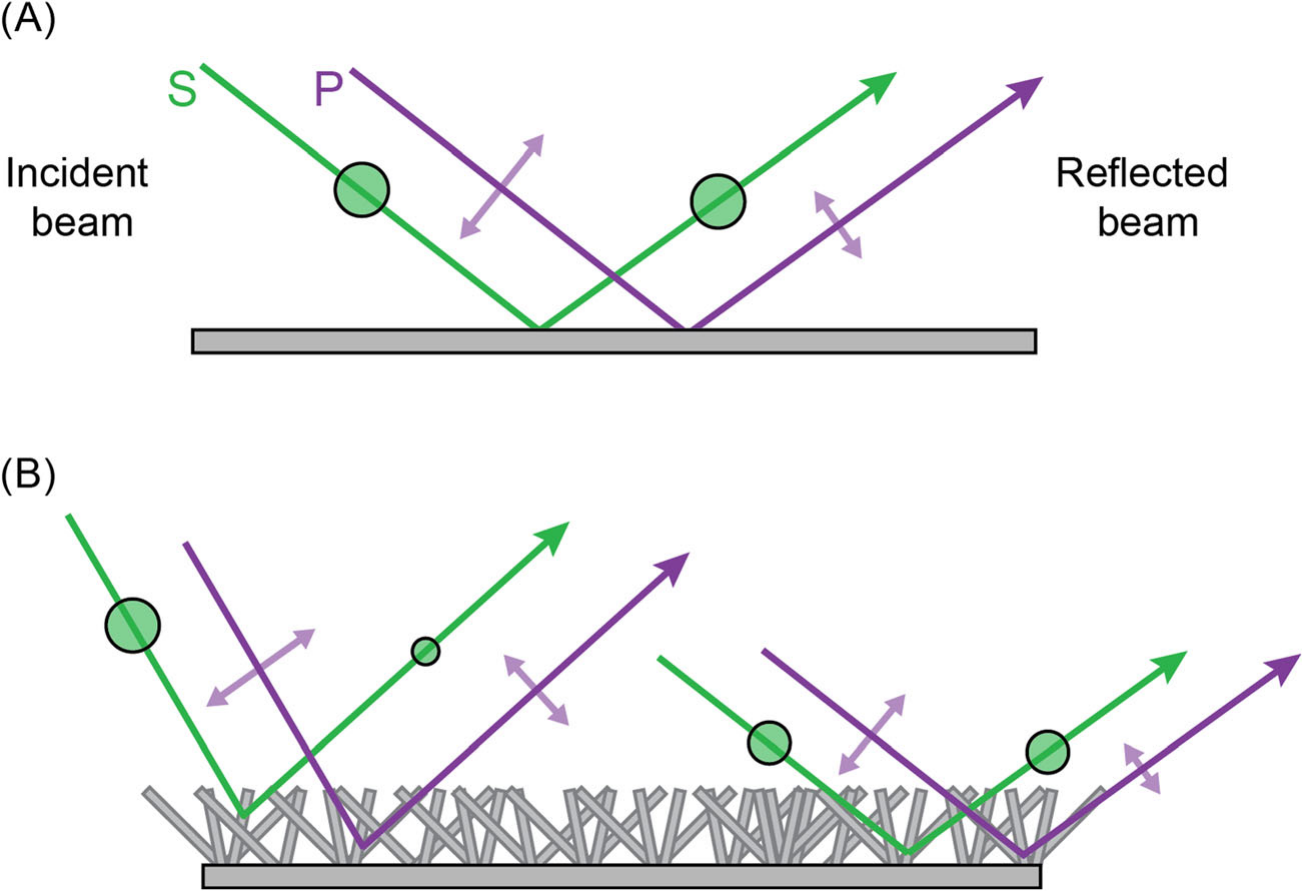
Comparison of reflected intensity of p- and s-polarised beams from a smooth (**a**) or fibrous material (**b**). **a** While a relatively large proportion of the intensity of s-polarised light (AoP into the page; intensity shown as size of the green circle) is preserved as the angle of reflection approaches Brewster’s angle (see Specular Reflections), reflected intensity of p-polarised light (AoP in the plane of the page; intensity shown as arrow length) decreases as a function of increasing angle of incidence, reaching a minimum at Brewster’s angle. **b** The same effects also occur in ‘rough’ or fibrous surfaces, but an observer at a given angle to the surface sees light that has been reflected at a greater diversity of angles, including those angles for which intensity of reflected p-polarised light is high (left beam)

Some more recent studies have controlled for reflected intensity cues by using surfaces that are matt, ‘rough’, or highly reflective (e.g. Mäthger et al. 2011; Calabrese et al. 2014; Melgar et al. 2015; Egri et al. 2016). These types of surface can help minimise the difference in intensity between s- and p-polarised reflections as a proportion of overall reflected intensity. Fibrous materials, such as felt and other fabrics, reflect and scatter incident light at a wide range of surface angles (Fig. 10). These two effects may be combined by using light-coloured fabric surfaces, with both a high diffuse reflectance and a high proportion of fibres oriented perpendicularly to the surface (Fig. 10).

To further decrease the relative contribution of direct specular reflections from a surface, bright, diffuse ambient lighting can also be added to an experimental arrangement. The apparent lightness of the surface can be manipulated through the ratio of the ambient- and stimulus-light intensities, provided that the surfaces illuminated have high diffuse reflectance. We propose that, through careful consideration of the materials visible to an animal during experiments and calibration of sources of ambient lighting, confounding cues from surface reflections can be essentially eliminated under most circumstances.

#### Viewing-angle effects in polarisers

**Fig. 11:**
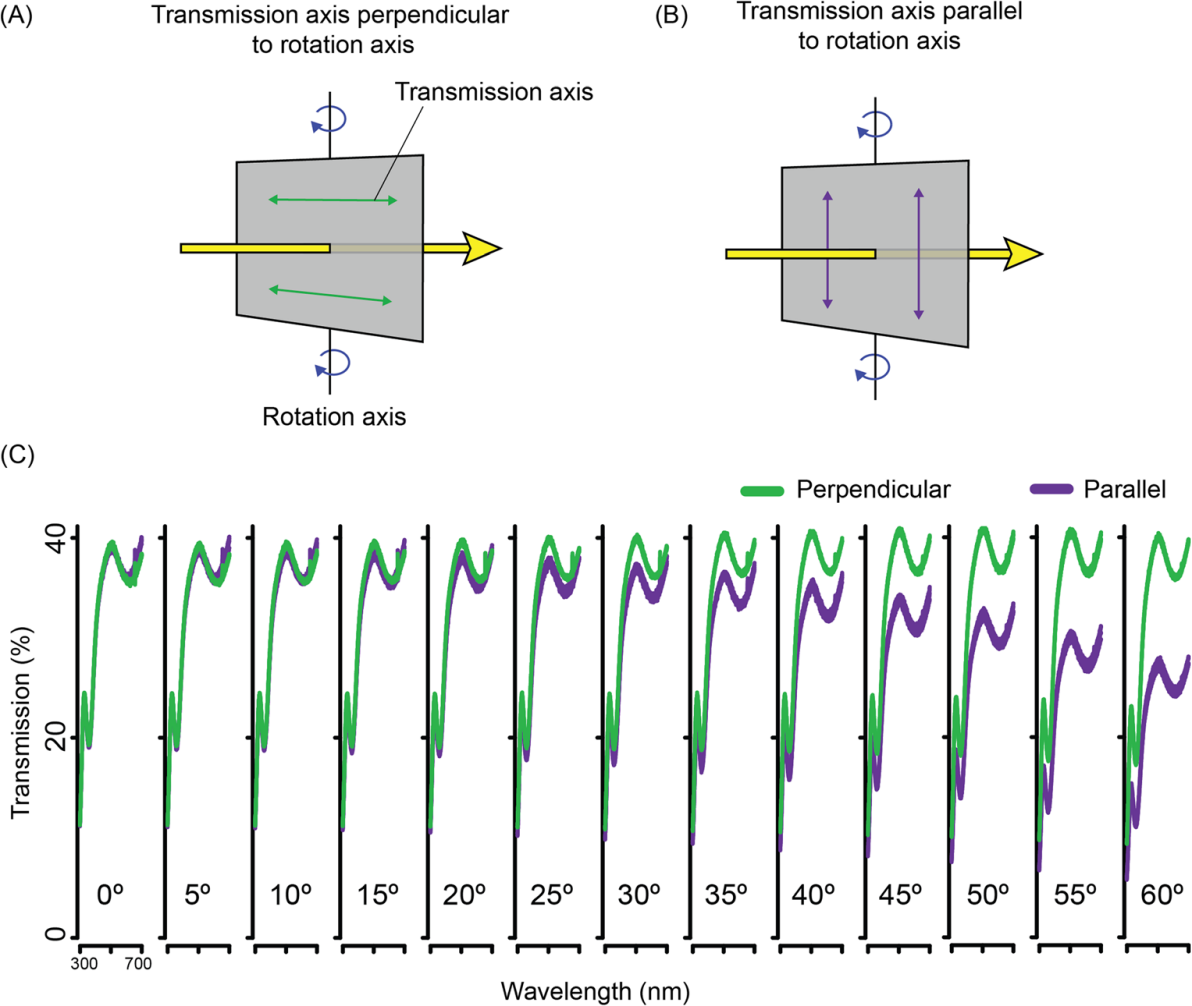
Changes in polarizer transmittance with angle of incidence. **a** The arrangement in which the polarizer’s transmission axis (TA) is perpendicular to the axis of rotation—relative to the incident beam (parallel to the plane of incidence). For this arrangement, transmission is not modulated as a function of angle of incidence. **b** The arrangement in which the polarizer’s TA is parallel to the axis of rotation. For this arrangement the proportion of the incident beam’s intensity that is transmitted decreases as a function of the angle of incidence (see panel **c**). **c** The transmittance spectrum of a UV-grade polarizer (HNP’B, Polaroid, USA) recorded at a range of incidence angles in the orientation described in (**a**, **b**). For incidence angles *≥* 25°, there is a clear reduction in transmitted intensity when TA orientation is parallel to the axis of rotation (as compared with when the TA is perpendicular to this axis). For more details of the measurement apparatus see supplement S6

**Fig. 12:**
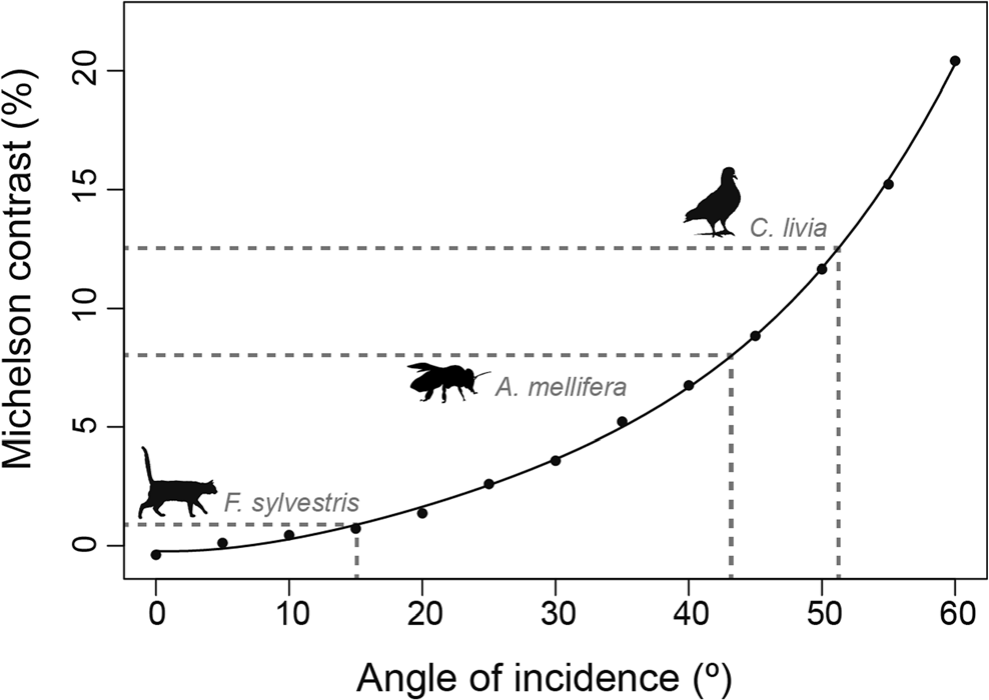
Intensity contrast between polarizer orientations. Michelson contrast in transmitted intensity between two adjacent polarizers with perpendicular transmission axes, at incidence angles 0°–60° (points: measured; line: fitted polynomial). Example maximum contrast sensitivity thresholds for model species (*Felis sylvestris*: Blake et al. 1974; *Columba livia*: Ghim and Hodos 2006; *Apis mellifera*; Bidwell and Goodman 1993) provided for reference

Laminated polarisers have been used to manipulate the AoP of stimulus light in most studies to date (see Sheet Polarisers). As a stimulus, an unpolarised beam transmitted through a polarizer should be the same intensity regardless of that polarizer’s transmission axis (TA) orientation. This basic requirement is met by all commercially available polarisers for a beam at normal incidence, but at non-normal incidence angles transmitted intensity varies as a function of TA orientation (Fig. 11; see also Wolf et al. 1980). As a result of this ‘viewing-angle’ effect, even at relatively modest off-axis incidence angles, differences in transmitted intensity may be discriminable. For incidence angles *≥* 40° the Michelson contrast (a measure of pattern detectability) between two polarisers with perpendicular TAs could be above the detection threshold (Fig. 12) for a range of animal species (e.g. cat *Felis sylvestris*: Blake et al. 1974; honeybee *Apis mellifera:* Bidwell and Goodman 1993) and above 50° this difference would be detectable to most species capable of spatial vision (e.g. goldfish *Carassius auratus*: Northmore and Dvorak 1979; various birds: Ghim and Hodos 2006; Lind et al. 2012). When two polarisers are presented to an animal simultaneously, the animal might therefore detect an intensity difference between them when viewing angle is not controlled.

**Fig. 13:**
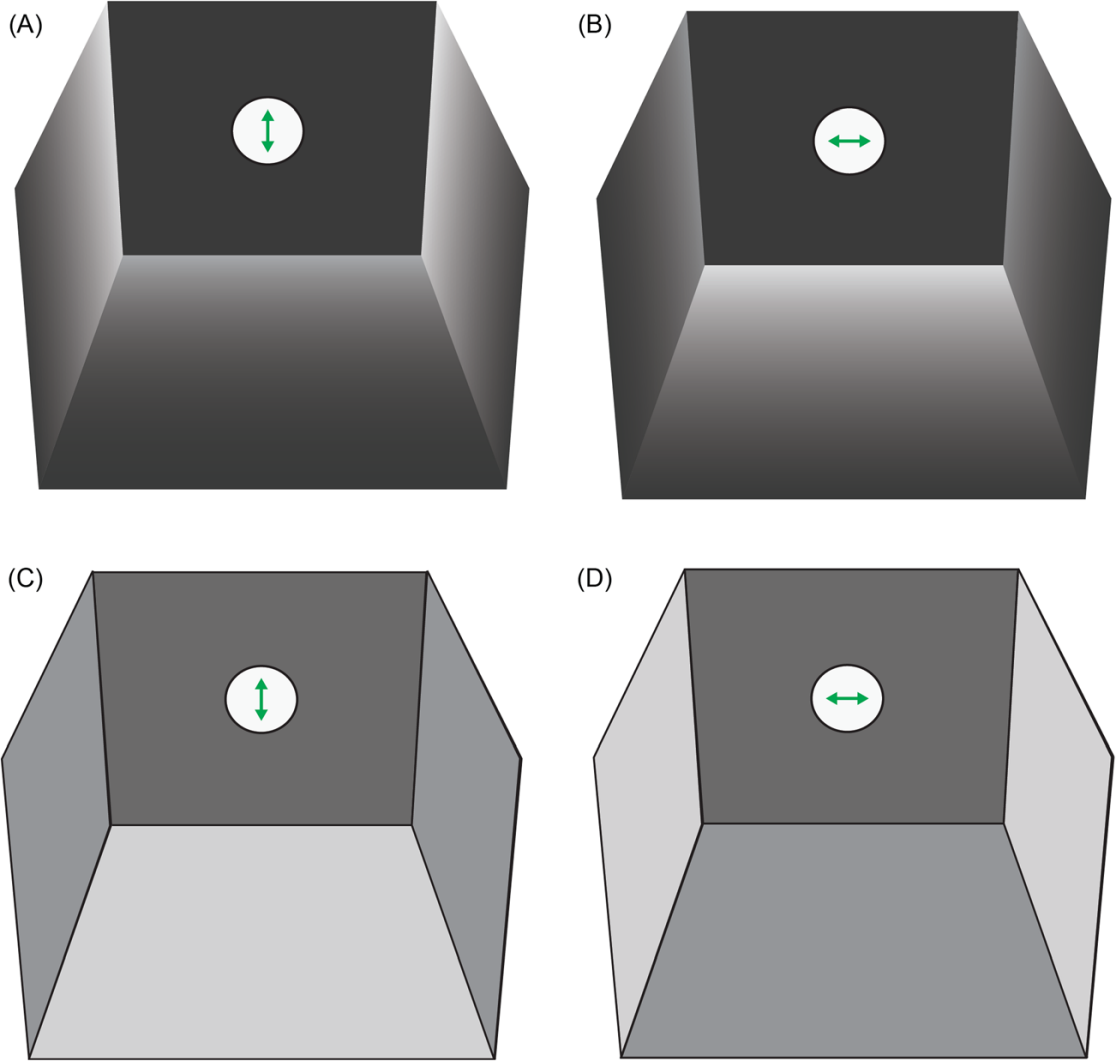
An illustrated example of a y-maze paradigm that might be confounded by polarised stimuli. In y-maze arm (**a**) vertically polarised stimulus light reflected from the chamber walls is brighter than that reflected from the floor. In arm (**b**) stimulus light is horizontally-polarised and the situation is reversed, making average brightness of the arm as a whole lower than for (**a**). In (**c**, **d**) the arena is lined with a rougher substance with a high diffuse reflectance, minimising these differences. In this scenario, projected off-axis transmitted illumination may act as an intensity confound. In y-maze arm (**c**) the transmission axis of the polarizer is vertical, and hence transmitted light that illuminates the chamber’s vertical walls is darker than that which illuminates the chamber’s horizontal floor. In y-maze arm (**d**) the polarizer’s transmission axis is horizontally oriented, and the pattern is reversed

In addition to observing changes in transmitted intensity directly, light transmitted at large incidence angles may also illuminate surfaces within an experimental arrangement. Surfaces illuminated at large incidence angles may receive more or less illumination as a function of TA orientation. When displaying a polarised stimulus, in the form of a backlit polarizer, in a box-shaped experimental chamber (such as a Skinner box, y-maze or choice-chamber experiment), projected off-axis transmission can form a confounding brightness pattern (Fig. 13). Thus, even when the animal’s viewing angle is restricted, intensity differences that result from transmission through a polarizer at large incidence angles may be present.

### Controlling for intensity confounds

#### Restriction of animal’s viewing angle

The effects discussed above can be addressed directly by restricting the angles at which the study animal views polarised stimuli and surfaces illuminated by them. In some cases it may be possible to measure the strength of surface-reflection cues across a range of angles, and the viewing angle effect for a given polarizer. These could be used to determine a range of viewing angles below the minimum discriminable contrast for the study species (see Fig. 12 for an example). This approach would, however, have limited applications for experimental paradigms requiring either broad-field stimuli or free movement of the study animal, and requires some prior estimate of contrast sensitivity.

#### Collimated stimulus light

**Fig. 14:**
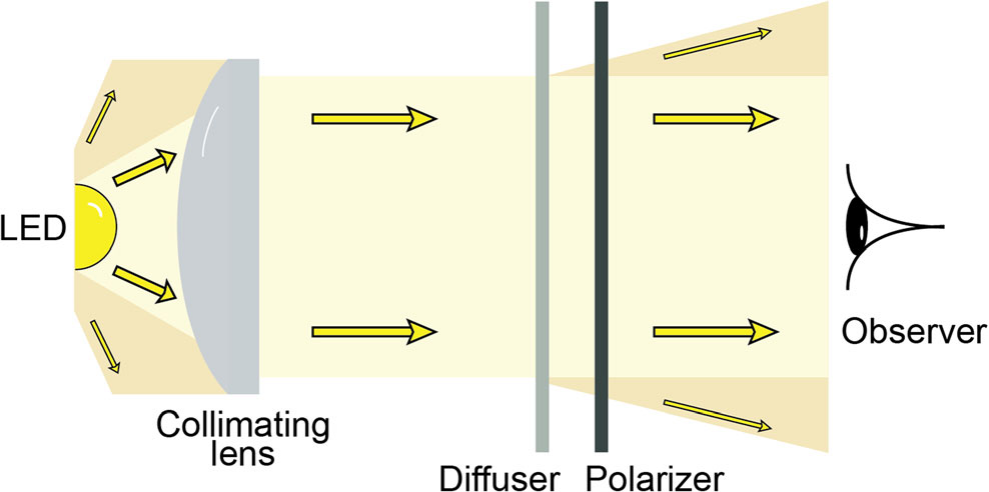
Collimating stimulus light. A collimating lens can be used to control the spread of a stimulus beam, so that the study animal observes stimulus light that is at normal incidence to the polarizer. Note that while some stimulus light is scattered by the diffuser, the spread is narrower than was the case for the initial light source

A simple means of limiting confounding intensity cues is to use a collimated light source. Collimated light may be produced via the introduction of a collimator, in the form of a lens or mirror, into the light path before the polarizer. The light beams then pass through the polarizer at close to normal incidence. This procedure has been used in a number of studies of polarisation sensitivity (McCann and Arnett 1972; Edrich and von Helverson 1976; Wolf et al. 1980; Henze and Labhart 2007), typically employing a light source–collimator–diffuser–polarizer light path (Fig. 14).

This method does, however, introduce some restrictions on animal position and light source properties. Because collimated light enters and exits the polarizer at a narrow range of viewing angles, the stimulus is not visible to the study animal outside of this range. Therefore this technique is better suited to electrophysiological investigations (e.g. McCann and Arnett 1972) or trackball behavioural paradigms (e.g. Henze and Labhart 2007) in which the position of the animal is necessarily restricted throughout the experiment.

Restrictions in light source properties may also be problematic. Standard practice is to introduce a diffuser directly before the polarizer, to ensure that light incident on the polarizer is not already polarised, which would modulate its intensity. Diffusers scatter normally incident light, reducing the effective collimation of light incident on the polarizer. In most cases, it may be practical to alter the order of optical components, so that the beam passes through the diffuser before the collimator.

## Summary

While technology progresses and new techniques are developed, it continues to be challenging to control for confounding cues when presenting polarised stimuli to animals. We aim to give the reader an overview of the most common and effective techniques currently available, and to provide a toolkit for critically assessing the suitability of these experimental methods in a research setting; although the methods for the production and calibration of polarised stimuli presented in this review necessarily represents only a subset of the full variety of available techniques.

The polarisation of light can be understood as several different properties of light, spatially and temporally averaged. Polarised light is abundant in nature, and it is the polarisation states of these light sources that are the most biologically relevant. The terminology used to describe the qualities of a polarised light beam varies somewhat within the biological literature, and we recommend that researchers clearly specify a definition and how it is calculated, for example using terms such as angle of polarisation (AoP) and degree of polarisation (DoP).

Polarised stimuli should be measured to understand their appearance to the animal under study. This requires some form of light detector and polarising filter, and can be achieved to different degrees of completeness across a given region in space and across the UV–visible spectrum; the most complete characterisation will often require more than one method. Measurement techniques should be chosen with reference to the aims of the experiment and what is known about the visual system of the study species.

A range of methods is available for controlling the angle and degree of linear polarisation of polarised stimuli. While the most commonly used methods, sheet polarizers and specularly reflecting surfaces, allow the production of stimuli that consist of a single AoP polarised to a high degree, recent advances make it possible to recreate more naturalistic and dynamic patterns of polarisation under controlled conditions.

Confounding cues are the predominant challenge in planning and conducting animal experiments involving polarisation. Their sources may be optical effects incidental to the design of the experimental apparatus, as well as insufficient control or calibration of the stimulus. We recommend a combination of control of the animal’s view of the stimulus, the stimulus’ properties themselves and careful measurement, as means to produce reliable, repeatable and meaningful results in polarisation vision studies.

## Electronic supplementary material

ESM 1 (PDF 2.14 MB).
