# Supplementary material for: Polarisation vision: overcoming challenges of working with a property of light we barely see
Source: Naturwissenschaften. 2018 Mar 27;105(3):27. doi: 10.1007/s00114-018-1551-3 (PMC5871655; doi:10.1007/s00114-018-1551-3)
Supplement: Supplementary file 1 — (PDF 2.14 MB). [file 114_2018_1551_MOESM1_ESM.pdf]

Supplement

Polarisation vision: overcoming challenges of working with a property of light we barely see

James J. Foster[*,a], Shelby E. Temple[b,†], Martin J. How[b], Ilse M. Daly[b], Camilla R. Sharkey[c], David Wilby[b] and Nicholas W. Roberts[b]

[a]*Vision Group, Department of Biology, Lund University, Sölvegatan 35, 223 62 Lund , Sweden*

[b]*Ecology of Vision Laboratory, School of Biological Sciences, Life Sciences Building, University of Bristol, Tyndall Avenue, Bristol, BS8 1TQ, UK*

[c]*Department of Physiology, Development and Neuroscience, Cambridge University, Cambridge, CB2 3EG, UK*

[*]*Corresponding author.* james.foster@biol.lu.se 0046462228629

[†]*Present Address: Azul Optics Ltd., 7 Bishop Manor Road, Westbury-On-Trym, Bristol, BS10 5BD, UK*

S1 Example Arrangement for Spectral Characterisation of Polarisation

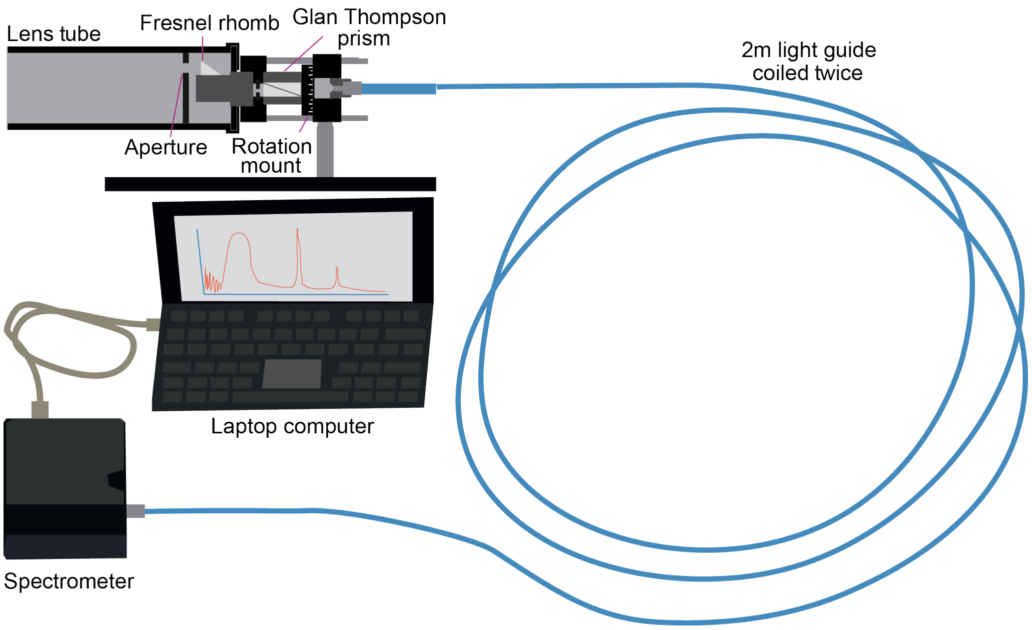

In this arrangement the beam of light is analysed by polarisers placed in the light path before it enters the optical fibre (blue). An aperture and lens tube restrict the region in space sampled, as well as the range of off-axis incidence angles at which light enters the polarisers. The Fresnel rhomb, which converts circular polarisation to linear polarisation, displaces the light path, so this displacement needs to be compensated for by raising (or lowering) the whole apparatus between the measurements of the Stokes parameters' linear (S_1 and S_2) and elliptical (S_3) components. Alternatively, an additional component can be added to allow the whole apparatus to rotate around the light-collecting surface of the Fresnel rhomb (Gagnon & Marshall, 2016). In this case, when the Fresnel rhomb's fast axis is aligned with the the Glan Thompson's transmission axis, the linear component is analysed, and when the Glan Thompson prism's transmission axis is at ±45° the elliptical component is measured.

Light relayed to the spectrometer by a coiled light guide should be depolarised, so no introduced polarisation affects measurements (see S2). Since only the ratio of the intensities recorded for each polariser orientation (see How Polarised Light is Measured) is required to calculate the polarisation state, rather than their precise radiometric values, the spectrometer does not need to be calibrated for spectral radiance measurements. Each measurement can be recorded as the number of spectrometer counts at each wavelength for a given integration time.

S2 Polarisation Sensitivity in a Spectrometer

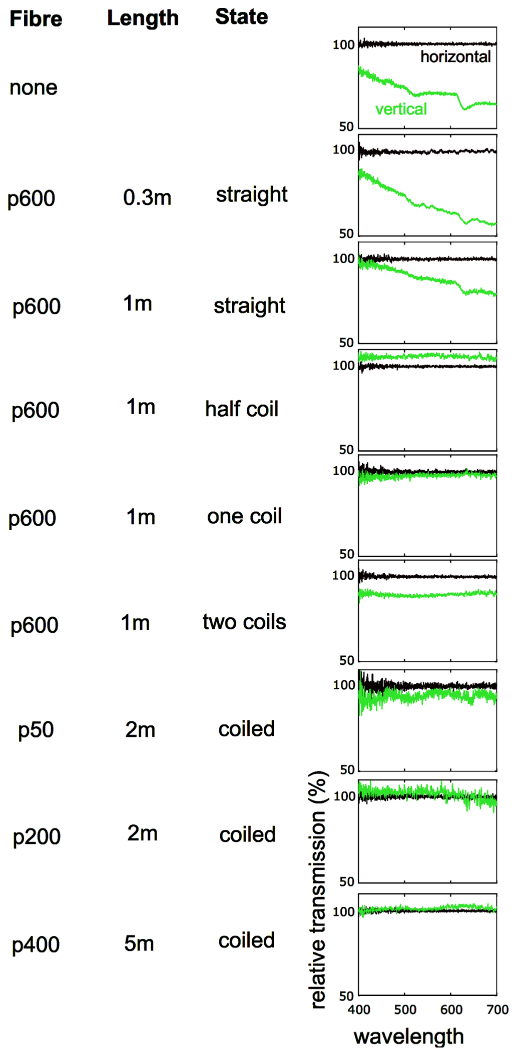

Due to optical effects such as mode propagation, it is possible that light with certain polarisation characteristics (*i.e.* specific angles) is propagated with less loss than other polarisation states. To check for this effect, record the intensity of light passing through a rotating linear polariser in front of an unpolarised light source with a bare fibre. The spectrum recorded by the device should remain constant regardless of the angle of incoming polarisation. Where polarisation sensitivity has not been eliminated, periodic fluctuations appear in the spectral measurement. This inherent polarisation sensitivity can be overcome by ensuring that a long (>1m) fibre is used and also by coiling the fibre multiple times. *N.B.* optic fibres are supplied with a recommended minimum bend radius, which should be avoided.

S3 Example Polariser Transmission Spectra and Prices

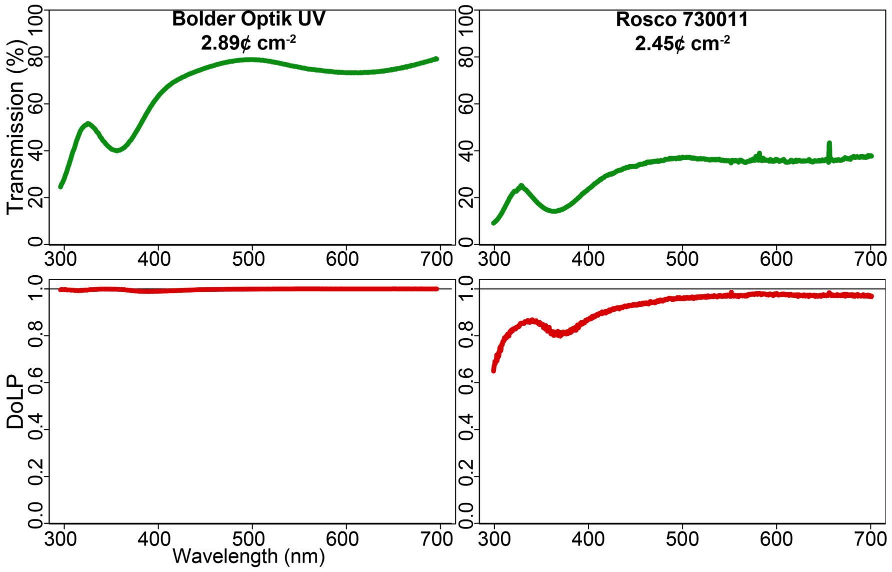

Transmission (**top**) and degree of linear polarisation (**bottom**) spectra for a technical grade (**left**: Bolder Vision Optik, USA) and theatrical grade (**right**: RoscoLab Ltd., UK) polarizing sheet. Prices in USD/100 per square centimeter based on unit prices for intact sheets (Bolder: 1000 × 620 mm; Rosco: 510 × 430 mm) excluding sales taxes and shipping costs.

S4 Retardation Devices—Retardance and Transmittance

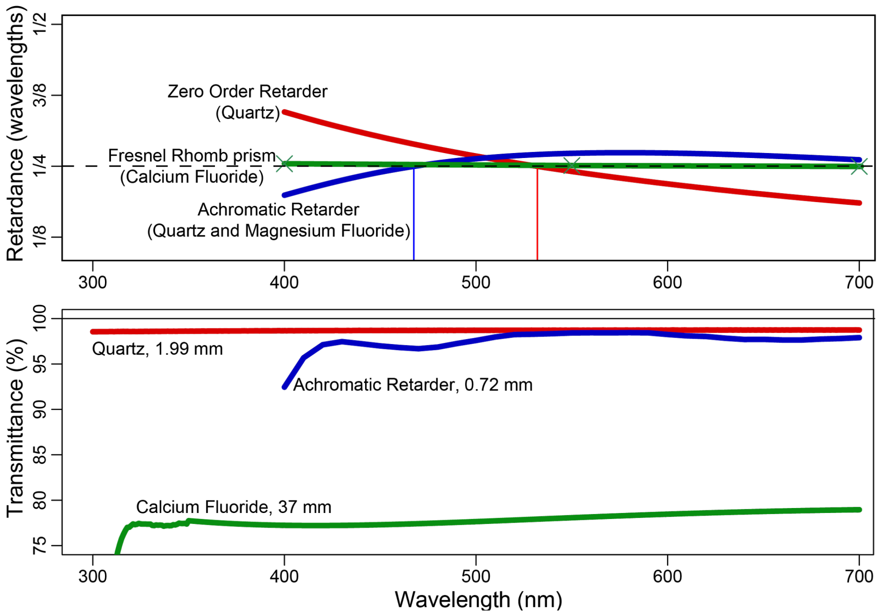

Retardance (**top**) and transmission (**bottom**) spectra for three types of optical-grade quarter wave retarder. Zero order retarders (red line; WPQ05M-532: Thorlabs GmbH, Germany) slow one component of linear polarisation by a given distance (across most of the spectrum) and therefore only retard by one quarter of a specific wavelength, as stated by the manufacturer (here 532 nm). 'Achromatic' retarders (blue line; AQWP05M-600: Thorlabs) retard the component parallel to their slow axis by approximately one quarter of a wavelength across a wider range. One advantage of zero-order and achromatic retarders is that they can be relatively thin, preserving the spectrum of transmitted light (**bottom**). Fresnel rhombs (green; FR600QM: Thorlabs; crosses: measured; line: fitted curve) retard by almost exactly one quarter

of a wavelength across their transmission spectrum, but the prism's shape requires the light path to be longer than for a zero-order or achromatic retarder with an equivalent collection area. All retardance measurements and material transmission spectra from www.thorlabs.com, transmittance spectra of individual retarders estimated from material transmission spectra and light path distance through the retarder for WPQ05M-532 and FR600QM.

S5 Measurements of Reflected s- and p-Polarisation.

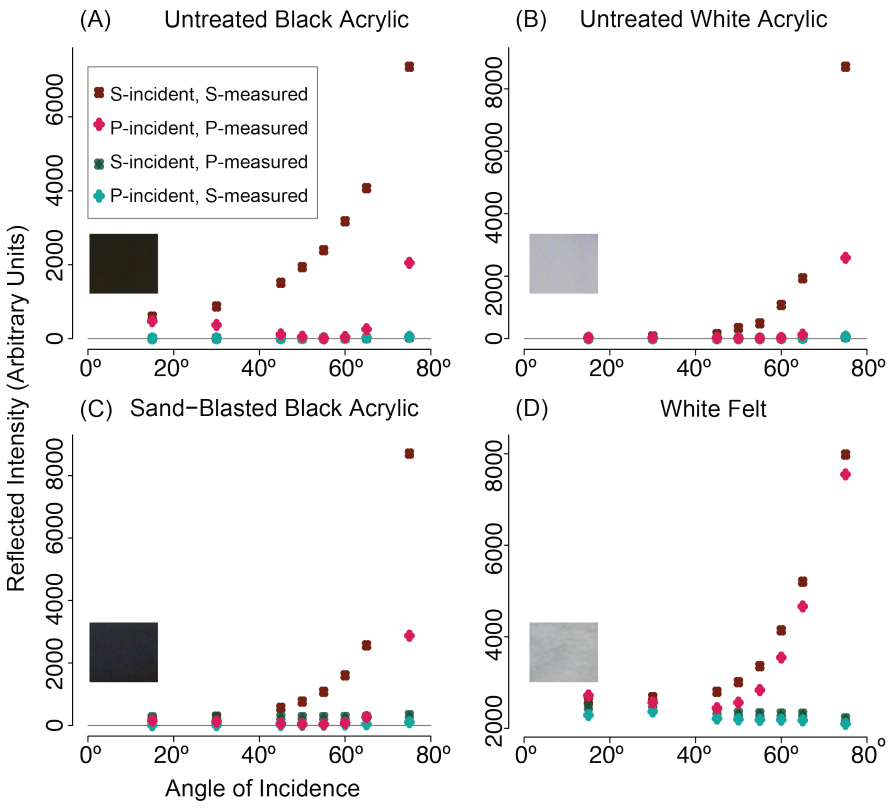

(**A**) Black acrylic (Perspex, Weybridge, UK). For angles of incidence 45–60° the intensity of p-polarised reflected light is near zero, as a proportion of the reflected intensity of s-polarised light. *N.B.* When incident light is s-polarised, none of its reflected intensity is p-polarised, and when incident light is p-polarised reflected light is not s-polarised.

(**B**) White acrylic (Perspex, UK). The ratio of reflected s-polarised light to p-polarised light is lower than for black acrylic.

(**C**) The same block of black acrylic as (A.), sand-blasted to create a 'rough' surface. The increase in ratio with angle of incidence is lower than for untreated black acrylic.

(**D**) White felt (Fabric Land Ltd., UK). This material is both highly reflective and 'rough' (fibrous), and hence the ratio of reflected s-polarisation to p-polarisation is low at all angles. Retardation by thin fibres may be responsible for the apparent conversion of s-polarised light to p-polarised, and p-polarised to s-polarised (green squares and rhombuses respectively). *N.B.* Reflected intensity falls within a narrower range in (D) than in the preceding panels.

S6 Goniometric Apparatus for Measuring Polariser Transmission.

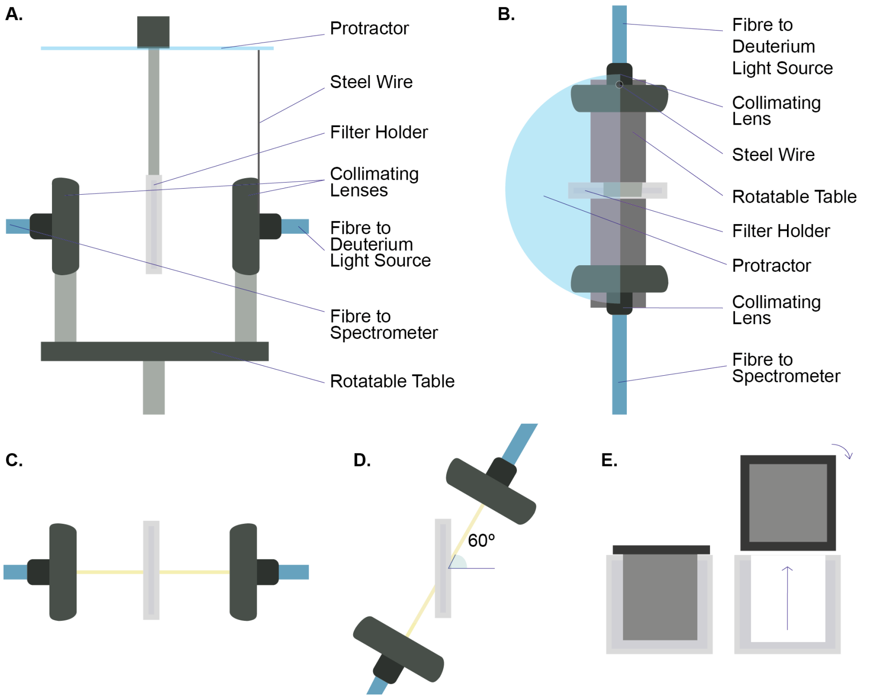

(**A**) A side view of the apparatus. **(B**) View of the apparatus from above. (**C**) Simplified view from above showing the light path (yellow) at an incidence angle of 0°. (**D**) The arrangement and light path for an incidence angle of 60°. (**E**) Front view of the filter holder, from which the polariser was removed and rotated by 90° between measurements.

In order to compare differences in transmission through two perpendicular TA orientations as a function of incidence angle, an apparatus was developed that allowed the projection of light through a polariser at a range of incidence angles. Broad spectrum light from a calibrated light source (DT-MINI-2-GS; Ocean Optics Inc., FL, USA) was projected through a collimating lens (74-UV; Ocean Optics Inc.), focussed to infinity, onto the polariser. Transmitted light was collected by another collimating lens, equidistant from the polariser, and relayed to a spectrometer (QE65000; Ocean Optics). Five sets of measurements across each incidence angle were recorded with the TA orientated perpendicular to the rotation axis (horizontal TA as viewed in (E)), with the TA oriented parallel to the rotation axis (vertical TA) and with no polariser (in order to calculate transmittance).

S7 Intensity artifacts at off-axis viewing angles in LCD monitors.

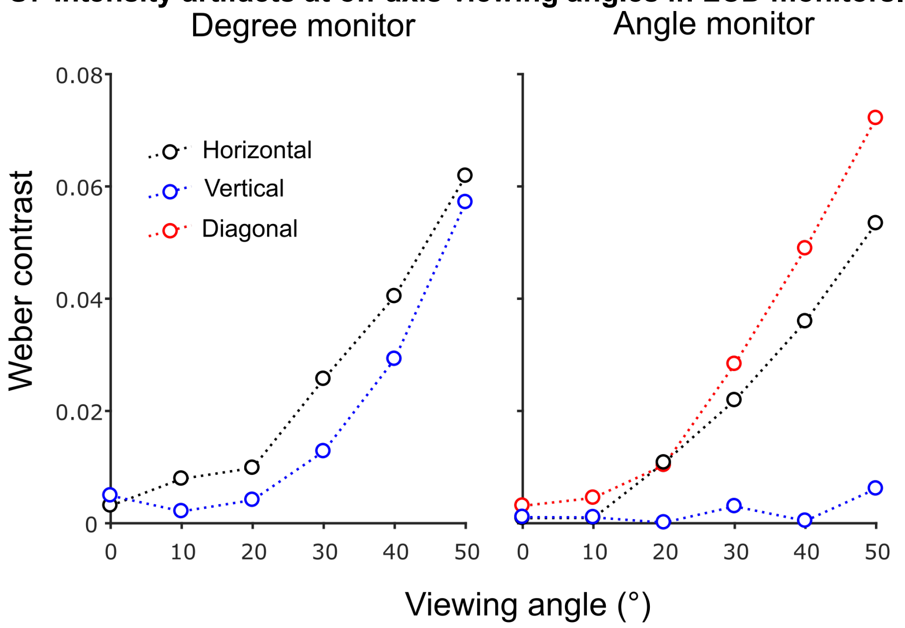

Contrast between the maximum and minimum voltage states (RGB values of 0 or 255; white or black in an unmodified LCD monitor) at off-axis viewing angles in the horizontal (black points) and vertical (blue points) planes, for an LCD monitor modified to control degree of polarisation (**left**; 1905FP, Dell) and one modified to control angle of polarisation (**right**; 1908fpc, Dell). Each measurement was taken through a coiled 2m, 400 μm diameter fibre with a ≤3° gershun tube , connected to a spectrometer (Flame, Ocean Optics). Since for the “angle” monitor the two states have ≈0 contrast for vertical off-axis viewing angles, a further set of measurements were made in the plane diagonal to the screen (red points).

S8 Calibration of LCD Monitors

As described in the main text, an LCD monitor may be converted to a polarisation monitor by removing the frontmost polariser, after which the polarisation of the monitor’s display may be controlled via a computer using the 256-level (8-bit) RGB scale. In order to understand how the specific brand and model of monitor alters the polarisation of transmitted light for different RGB values, it is absolutely essential to measure the angle and degree of polarisation as well as the ellipticity of light from the monitor at a particular RGB value using one of the methods described in How Polarised Light is Measured. The optical properties of the monitor can vary considerably as a function of wavelength and RGB value, so it may be necessary to measure polarisation across the spectrum and to sample at frequent intervals across the 8-bit range. Where wavelength dependence becomes problematic, it may be avoided by replacing the monitor’s backlight with a custom light source, such as a bank of LEDs with a narrow emission spectrum.Once steps have been taken to avoid RGB values between which polarisation varies excessively, an experimenter may present an animal with a complex dynamic stimulus composed of regions with different polarisation characteristics with little to no intensity variation.

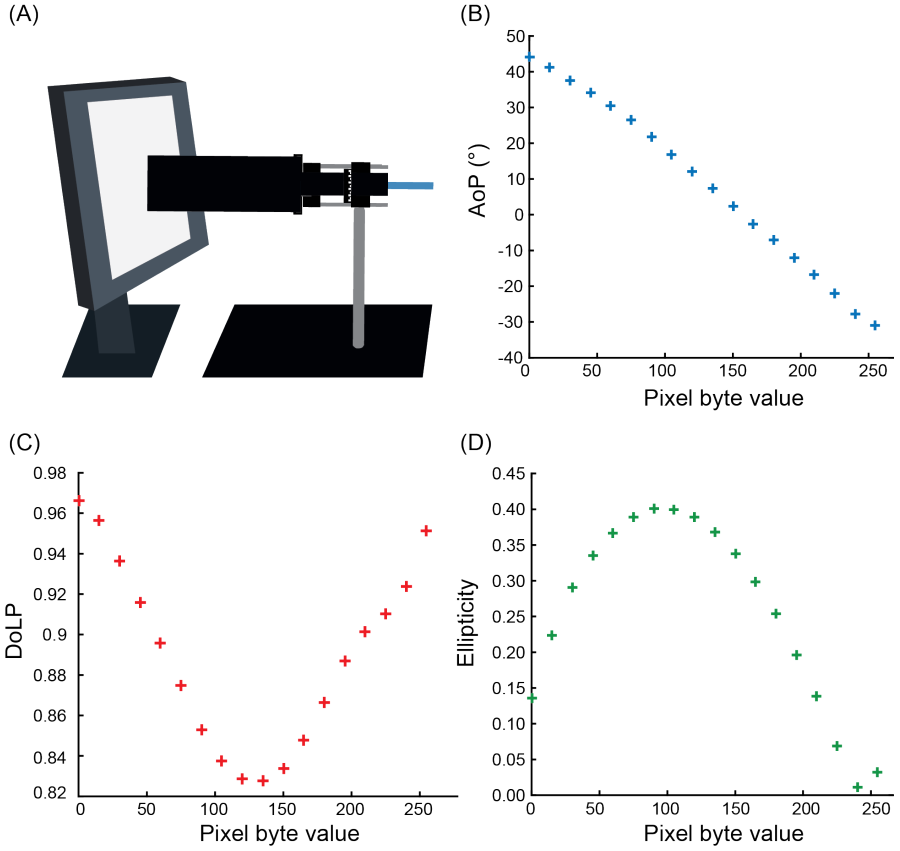

Measurement and calibration procedure for an LCD monitor. (**A**) Schematic showing arrangement of polarimetry equipment. (**B–D**) Examples of measurements for each pixel byte (RGB) value. The change in angle and the degree of polarisation across the 256-level greyscale on a modified TN-film (AoP) monitor, (VL-15EC6, Viglen ltd., UK). This monitor can produce a ≈80° range of angles of polarisation. At pixel byte values 0 and 210, angle of polarisation differs by ≈78°, while remaining close in degree of linear polarisation (**C**), so these two values could be chosen to create a pattern contrasting in angle of polarisation.
